# An overview of systematic reviews on the collaboration between physicians and nurses and the impact on patient outcomes: what can we learn in primary care?

**DOI:** 10.1186/s12875-017-0698-x

**Published:** 2017-12-22

**Authors:** Evi Matthys, Roy Remmen, Peter Van Bogaert

**Affiliations:** University of Antwerp, Campus Drie Eiken DR334, Universiteitsplein 1, 2610 Wilrijk, Belgium

**Keywords:** Nurse, Physician, Primary care, Collaboration, Inter-professional, Patient outcome, Education

## Abstract

**Background:**

Primary care needs to be strengthened in order to address the many societal challenges. Group practices in primary care foster collaboration with other health care providers, which encourages care co-ordination and leads to a higher quality of primary care. Nursing roles and responsibilities expanded over time and nurses have been found to often provide equal high-quality chronic patient care compared to physicians, even with higher patient satisfaction. Inter-professional collaboration between primary care physicians and nurses is a possible strategy to achieve the desired quality outcomes in a strengthened primary care system. The objective of this research is to synthesize the evidence presented in literature on the impact of collaboration between physicians and nurses on patient outcomes in primary care or in comparable care settings.

**Methods:**

A systematic review of peer-reviewed reviews was performed in four databases: COCHRANE, MEDLINE, EMBASE and CINAHL. All studies from 1970 until May 22 2016 were included in the search strategy. Titles, abstracts and full texts were respectively reviewed. At least two of the three authors independently reviewed each of the 277 abstracts and 58 full texts retrieved in the searches to identify those which contained all the inclusion criteria. Two authors independently appraised the methodological quality of the reviews, using the AMSTAR quality appraisal tool.

**Results:**

A total of eleven systematic reviews met all the inclusion criteria and almost fifty different patient outcomes were described. In most reviews, it was concluded that nurses do have added value. Blood pressure, patient satisfaction and hospitalization are patient outcomes where three or more systematic reviews concluded better results when physicians and nurses collaborated, compared to usual care. Colorectal screening, hospital length of stay and health-related quality of life are outcomes where collaboration appeared not to be effective.

**Conclusions:**

Collaboration between physicians and nurses may have a positive impact on a number of patient outcomes and on a variety of pathologies. To address future challenges of primary care, there is a need for more integrated inter-professional collaboration care models with sufficiently educated nurses.

**Electronic supplementary material:**

The online version of this article (10.1186/s12875-017-0698-x) contains supplementary material, which is available to authorized users.

## Background

Populations around the world are rapidly ageing. It is estimated that between 2015 and 2050, the world’s population of over 60 year olds’ will nearly double from 12 to 22% [1]. As people age, they are more likely to experience several health conditions at the same time. The demand for health care is evolving rapidly in the context of an ageing population and the growing number of people living with one or more chronic conditions [2]. In Europe, patients are more demanding and expect health care to be accessible and high qualitative at the same time [2, 3]. Professional caregivers, on the other hand, experience a high workload and demand a better work-life balance [4, 5]. At the same time, financial resources in health care are decreasing, while the demand for financial support is increasing [6–8]. In an attempt to address these challenges, the following four aims have the potential to guide innovations in health care delivery: improving the health of populations, improving the experience of care, reducing per capita costs of health care, and diminishing the workload for professional caregivers so they can rediscover meaning and joy in their work [7, 9, 10].

Reforms are shifting care from hospitals to community, partly due to a growing prevalence of chronic diseases [11, 12]. In addition, countries in the European Union show many potentially avoidable hospital admissions for several chronic conditions including diabetes mellitus, chronic heart failure, chronic obstructive pulmonary disease and asthma. Potentially avoidable hospitalizations for these conditions are commonly used to measure access and quality of primary care systems [13, 14]. In order to address the needs of ageing populations and to reduce the unnecessary use of hospital care, primary care systems should be strengthened [2].

It was suggested that group practices in primary care foster collaboration with other health care providers, which encourages care co-ordination and leads to a higher quality of primary care [8]. Primarily, nurses were introduced in primary care practices to meet a perceived shortage of primary care physicians [15]. Over time, nursing roles and responsibilities expanded. Practice nurses were able to provide holistic care for patients that was not limited to traditional nursing boundaries [16]. Nurses have been found to often provide cost effective patient care and equal high-quality chronic patient care compared to primary care physicians, even with higher patient satisfaction [2, 12, 16, 17]. By expanding the roles and responsibilities of nurses, primary care systems can be strengthened.

Improved inter-professional collaboration is important and diversity of disciplines is needed in a time when the provision of primary health care becomes more complex and one health professional can no longer meet all patient needs [18, 19]. As the largest health care workforce group, and because of their specific skills and competencies, nurses are in an ideal position to collaborate with other team members in the delivery of more accessible and effective chronic disease management in primary care. Inter-professional collaboration between primary care physicians and nurses is a possible strategy to achieve the desired quality outcomes in an effective and efficient manner in an integrated health system. Therefore, there’s a need to explore to what extent an integration of physician and nurse competencies impacts patient outcome.

The objective of this research is to synthesize the evidence presented in literature on the impact of collaboration between physicians and nurses on patient outcomes in primary care or in comparable care settings.

## Methods

### Data sources

We searched for reviews of the literature containing synthesized evidence relating to collaboration between physicians and nurses, and the impact of their collaboration on patient outcomes.

Searches were performed in four literature databases: COCHRANE, MEDLINE, EMBASE and CINAHL. All databases were searched from 1970 (or from their inception if this was later than 1970) until May 2016. In addition, reference lists of the selected reviews were reviewed to identify other eligible reviews, but no additional review articles were identified.

All detailed search strategies can be found in Additional file 1.

The retrieved references were entered into Endnote© and duplicates were removed.

### Study selection

The included studies had to fulfil a number of criteria in order to be included. First, the manuscript had to be a systematic review of the literature. A review was considered a systematic review if two of the following criteria were met: a search strategy was reported, a search was performed in Medline(PubMed) at least, and the included studies were subjected to a methodological assessment. There were no inclusion criteria based upon the research design of the primary research articles included in the systematic reviews.

Second, the manuscript needed to concern ‘collaboration between physicians and nurses’ in a primary care setting or in a hospital setting. Since there is no generally accepted definition of what inter-professional collaboration means, the intervention was defined as collaboration by the researchers if at least one physician provided care along with at least one nurse.

Third, the outcomes in the reviews needed to concern clinical patient outcomes and/or patient satisfaction outcomes. The review also needed sufficient methodological quality according to the AMSTAR quality appraisal tool (studies with a score ≥ 11 were included) [15–17]. And finally, none of the exclusion criteria listed below were met.

Research publications were excluded when they were primary research studies, when they were written in a language other than English or Dutch, or when the setting was considered ‘inappropriate’. Settings were defined as inappropriate when the presented patient population was dissimilar or incomparable to the primary care population. Inappropriate settings were determined as; an intensive care unit (ICU), radiology, neonatology intensive care unit (NICU), obstetrics and gynecology. Studies were also excluded when the outcomes merely concerned nurse/physician outcomes.

A four-stage inclusion process was applied. Initially, titles and abstracts of research articles identified from the search strategies were screened, in order to determine their relevance and whether they met the inclusion criteria. No further analysis was done on the subsequent criteria as soon as one criterion was not met. In the first stage, one reviewer screened all references. When the title provided insufficient information to determine inclusion or exclusion, the research article proceeded to the second stage.

In the second stage, two reviewers independently examined all abstracts of the articles selected in the first stage, in order to determine whether they met the inclusion criteria. Any disagreements were resolved by discussion between the two reviewers.

In the third stage, two reviewers independently examined all full texts of the articles selected in the second stage. Any disagreements were resolved by discussion between the two reviewers. If no agreement could be reached, a third reviewer decided.

The final stage of inclusion related to the methodological assessment of the reviews. All reviews remaining after the third stage, were assessed with the AMSTAR quality appraisal tool [15, 17]. This assessment tool was formed by combining the enhanced Overview Quality Assessment Questionnaire (OQAQ), a checklist created by Sacks, and three additional items judged to be of methodological importance. 11 different components were identified [15]. The eleven criteria were scored as followed: 2 points were given when the criterion was fully met, 1 point when it was partly met and zero points when it was not met. Therefore, a maximum of 22 points on methodological quality could be achieved (see Table 2). Two reviewers independently examined the methodological quality of the reviews, using the AMSTAR quality appraisal tool [18]. The mean of the scores of the two reviewers was computed and classified as the final quality score [17]. In case the scores of the reviewers differed more than two points, reviewers reached consensus by discussion. Only moderate and high quality reviews (mean scores ≥11) were used for data extraction.

### Data-analysis and synthesis

Data were extracted about the search strategies, time frame of the searches, studied interventions, selected outcomes, selected patient populations, selected study setting, the collaboration between physician(s) and nurse(s) and the different nursing roles within the collaboration.

Data-analysis was done primarily by description of the characteristics, interventions and outcomes. Meta-analyses and quantitative assessments from the included reviews were described. No quantitative pooling was performed across the reviews.

## Results

### Search and inclusion results

**Fig. 1:**
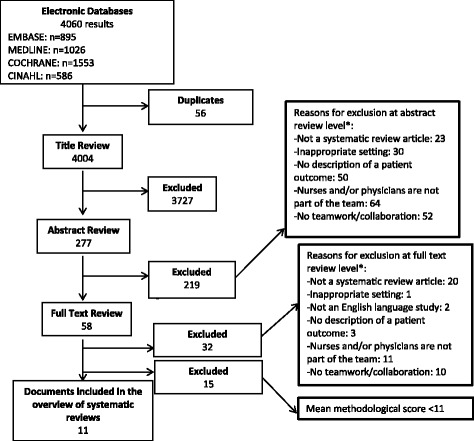
Search strategy. Presents the search strategy of this overview of systematic reviews. The reasons for exclusion after reviewing the abstracts and full texts are presented on the right. *Reasons for study exclusion can be attributable to more than one category

After duplicates were removed, the searches in the different databases resulted in one unique database, encompassing 4004 studies. Titles, abstracts and full texts were respectively reviewed and subsequently 277 studies and 58 studies were identified as potentially meeting the inclusion criteria (See Fig. 1). A total of 36 systematic reviews met all the inclusion criteria. Two reviewers independently assessed the remaining 36 reviews on their methodological quality, using the AMSTAR quality appraisal tool. A mean of the two scores was computed and classified as the final quality judgement. Eleven systematic reviews had a mean quality score higher than 11 and were included for data-extraction and analysis.

The flow diagram of the inclusion process is shown in Fig. 1.

Characteristics of the 11 included systematic reviews.

Search periods for each systematic review are shown in Table 1.

**Table 1:** Search periods in included review articles

Review article	Search period
Allen et al. 2014	1990–2013
Aubin et al. 2012	1947–2009
Health Quality Ontario. 2013	Inception-2012
Health Quality Ontario. 2014	2000–2013
Martin et al. 2010	1999–2009
Newhouse et al. 2011	1990–2008
Renders et al. 2000	1966–1999
Shaw et al. 2014	1980–2014
Smith et al. 2014	1990–2011
Snaterse et al. 2016	1990–2015
Stalpers et al. 2015	2004–2012

A narrative overview of the included review articles is described in Table 2. The eleven reviews only included quantitative studies. Four reviews [19–22] were limited to randomized controlled trials only, while the other seven reviews also included other comparative designs such as controlled before and after studies, interrupted time series and intervention studies. Three reviews included observational studies [23–25]. One review author additionally included other systematic reviews [26].

**Table 2:** Characteristics of the systematic reviews (*n* = 11)

Author, country, year	Objectives	Design	Patient population + setting	(Patient) Outcomes	Results/Conclusions	Quality assessment
1. Allen et al., Australia, 2014	To locate and synthesise research using randomized control trial designs on quality of outcomes following transitional care interventions compared with standard hospital discharge for older people with chronic illnesses. To make recommendations for research and practice.	Systematic review. 12 quantitative studies included: - 12 randomized controlled trials	- Older patients diagnosed with chronic illnesses - Transition from acute hospital care to (nursing) home	- Length of hospital stay - Length of time till re- hospitalization - Length of re- hospitalization - Costs - Functional status - Depression - Patient satisfaction - Quality of life - General practitioner (GP) satisfaction	Collaboration between nurses and physicians in the ‘Discharge protocol and advanced practice nurse’ intervention: - Delay in re- hospitalization. - Fewer days of re- hospitalization. - Fewer days of hospitalization. - Lower costs. - No significant difference in functional status, depression or patient satisfaction. Collaboration between nurses and physicians in the ‘General practitioner and primary care nurse models’ intervention: - Mixed results for (re- )hospitalization. - Higher patient satisfaction. - Improved referral to community services. - Higher GP satisfaction. - Faster discharge communication to GPs. General practitioner and practice nurse interventions were not effective in the reduction of hospitalization rates or length of stay. Low response rate of general practitioners makes interpretation of the results difficult.	19
2. Aubin et al., Canada, 2012	To describe and classify the various interventions studied in the literature to improve continuity of care in the follow- up of patients with cancer. To determine the effectiveness of interventions aiming to improve continuity of cancer care, on patient, healthcare provider and process outcomes.	Systematic review and meta- analysis. 51 quantitative studies included: - 49 randomized controlled trials - 2 controlled clinical trials (5 studies had a multi- disciplinary approach as intervention)	- Patients (65 years and older) with a cancer diagnosis - Patients admitted to the hospital with a terminal prognosis of 2 weeks to 6 months - Patients diagnosed with cancer and receiving Medical oncology outpatient clinic - Hospital setting	Patient outcomes: - Quality of life - Functional status - Physical performance - Pain - Depression - Anxiety - Satisfaction - Survival - Emotional adjustment - Cognitive functioning Informal caregiver outcomes Process outcomes	Three out of the five studies assessing interdisciplinary team models of care reported significant improvements in one or more classes of patient health- related outcomes during the study follow- up period. Patients supported by the multidisciplinary specialist Palliative Care Team had: - Significant improvements in scores of symptom severity. - A significantly better mood and were less bothered by emotional problems. - Significantly better quality of life scores at one and four weeks of follow- up, compared to patients assigned to the control group. Based on the median effect size estimates and the 95% CI, no significant difference in patient health measures was found. According to the descriptive analysis of single interventions on the improvement of patient health- related outcomes, case management and interdisciplinary teams seemed to be the most favourable models of care to improve one or more classes of patient outcomes.	20
3. Health Quality Ontario, Ontario, 2013	To determine the effectiveness of specialized nurses who have a clinical role in patient care in optimizing chronic disease management among adults in the primary health care setting.	An evidence based analysis. 6 quantitative studies included: - 6 randomized controlled trials (8 papers)	- Patients with a chronic disease(s)/type 2 diabetes/asthma/hypertension/dementia/chronic obstructive pulmonary disease (COPD)/cancer/coronary artery disease (CAD)/congestive heart failure (CHF) - Primary health care setting - General internal medicine clinic	- Hospitalizations - Length of stay - Mortality - Emergency department visits - Specialist visits - Health- related quality of life (HRQOL) - Patient satisfaction - Disease- specific measures - Process measures - Examination or medication prescribing - Health- system efficiencies - Number and length of primary health care visits - Physician workload	Specialized nurses working with physicians showed a general increase in process measures related to clinical examinations and medication management based on guidelines. - Significant reduction in HbA1c among diabetes patients. - Significant increase in the proportion of CAD patients with controlled BP and total cholesterol. - Significant reduction in hospitalization after 1 year for CAD patients receiving secondary prevention. - More patient satisfaction with care provided by the nurse plus physician intervention. - Inconsistency regarding outcomes related to HRQOL. No outcomes indicated specialized nursing interventions to be more harmful than physicians alone. Unclear role of the specialized nurse, making it difficult to determine the impact on efficiency. More research is needed to better understand the impact of specialized nurses on primary health care efficiency.	15
4. Health Quality Ontario, Ontario, 2014	To systematically review team- based models of care for end- of- life service delivery, to determine whether an optimal model exists.	Systematic review and meta- analysis. 12 quantitative studies included: - 2 systematic reviews - 10 randomized controlled trials	- Patients (adults) with advanced diseases (cancer, dementia, organ failure, stroke, chronic heart failure) receiving end of life care. - Home care - Hospital care	- Patient quality of life - Symptom management - Patient satisfaction - Informal caregiver satisfaction - Health care provider satisfaction - (Nursing) home death - Advance care planning - Emergency department visits - Hospital/ intensive care admission - Hospital length of stay	The review considered the core model components of team membership, services offered, mode of patient contact, and setting. Team membership includes at minimum a physician and nurse, one of who is specialized in end- of- life health care. Team services included: symptom management, psychosocial care, and development of patient care plans, end- of- life care planning, and coordination of care. Comprehensive team- based model: moderate- quality evidence that a comprehensive team- based model with direct patient contact significantly: - Improves patient QOL, symptom management and patient and informal caregiver satisfaction. - Increases the patient’s likelihood of dying at home. - Decreases the patient’s likelihood of dying in a nursing home. - Has no impact on hospital admissions or hospital length of stay. Hospital team- based model: no impact on length of hospital stay, significant reduction of ICU admissions. Home team- based model: significantly increases patient satisfaction, increases the patient’s likelihood of dying at home and significantly decreases emergency department visits and hospital admissions. Findings are applicable to deliver care to people with an estimated survival of up to 24 months.	16
5. Martin et al., Switzerland, 2010	To provide an overview of the evidence base for inter- professional collaboration and new models of care in relation to patient outcomes.	A qualitative synthesis. 14 quantitative studies included: - 14 randomized controlled trials	Elderly with: - acute/chronic diseases - risk factors - Patients after stroke - Patients with hip fracture/type 2 diabetes/Alzheimer disease/ chronic heart failure/multi- morbidity/problems in cognition, activities of daily living (ADL) - Children with asthma - Patients with bipolar disease/depression - Primary care - Hospital setting - Outpatient clinic	- Mortality - Clinical outcomes - Functional outcomes - Social outcomes - Utilisation of medical services - Patient- reported outcomes: QOL, ADL and satisfaction with care.	Mixed results were reported regarding: - Mortality. - Physical, emotional and social functioning. - Utilisation of medical services. - Hospitalization rates and length of hospital stay. Patient reported outcomes: significantly higher score of self- perceived health and life satisfaction. Mixed results regarding activities of daily living. Four studies showed that participants who experienced collaborative care management models were significantly more satisfied with their care than usual- care recipients. The evidence base of inter- professional collaboration shows promising results in relation to patient outcomes.	13.5
6. Newhouse et al., USA, 2011	Compared to other providers (physicians or teams without advanced practice registered nurses (APRN)), are APRN patient outcomes of care similar?	Systematic review. 69 quantitative studies included: - 20 randomized controlled trials (RCTs) - 49 observational studies	- Pregnant women - Neonates - New- borns - Children - Adults - Elderly - Community - Primary care - Inpatient - Nursing home - Ambulatory - Surgery - Prenatal- inpatient - Hospital	- Patient satisfaction - Self- reported perceived health - Functional status - Glucose control - Lipid control - Blood pressure (BP) - Emergency department/urgent care visits - Hospitalization - Duration of mechanical ventilation - Length of stay - Mortality - Cost - Complication	37 studies examined patient outcomes of care by nurse practitioners (NP care group) compared with care management exclusively by physicians. - High level of evidence to support equivalent levels of patient satisfaction, self- reported perceived health, functional status outcomes, glucose control and BP control. - High level of evidence to support equivalent rates of emergency department visits, hospitalization and mortality. - High level of evidence to support better serum lipid levels. - Moderate level of evidence to support equivalent length of stay. 11 studies examined clinical nurse specialist (CNS) outcomes. - High level of evidence to support equivalent patient satisfaction scores. - High level of evidence to support equivalent or lower length of stay for patients cared for in the CNS group. - High level of evidence to support the CNS group has a lower cost of care High level of evidence that APRNs provide safe, effective quality care to a number of specific populations in a variety of settings. APRNs have a significant role in the promotion of health in partnership with physicians and other providers,	17.5
7. Renders et al., Amsterdam, 2000	To determine the effectiveness of different interventions, targeted at health care professionals or the structure in which health care professionals deliver their care, to improve the care for patients with diabetes in primary care, outpatient and community settings.	Systematic review. 41 quantitative studies included: - 27 randomized controlled trials - 12 controlled before and after studies - 2 interrupted time series	- Non- hospitalised patients with Type 1 or Type 2 diabetes mellitus. - A primary care setting - Outpatient (ambulatory care) setting - Community setting (managed care organisations, general medical clinics)	- Glycaemic control - Micro- or macro- vascular complications - Cardiovascular risk factors - Hospital admissions - Mortality - Well- being - Perceived health - Quality of life - Functional status - Patient satisfaction	The addition of patient education or a more enhanced role of a nurse to a complex intervention strategy seems to be important to improve patient outcomes besides process outcomes. Nurses can play an important role in facilitating compliance or giving patient education. They can even replace physicians in delivering many aspects of diabetes care, if detailed management protocols are available, or if they receive training. The seven studies in which nurses replaced (partly) physicians in providing diabetes care generally demonstrated a positive impact on glycaemic control. The effectiveness of the implementation of revision of professional roles as a single intervention remains unclear.	21
8. Shaw et al., USA, 2014	To synthesize the current literature describing the effects of nurse- managed protocols, including medication adjustment, for the outpatient management of adults with common chronic conditions, namely diabetes, hypertension and hyperlipidaemia.	Systematic review and meta- analysis. 18 quantitative studies included: - 16 randomized controlled trials - 2 controlled before- and- after studies. (2 companion articles-methods or follow- up)	- Adults with elevated cardiovascular risk - General medical hospital - Specialty hospital - Primary care clinic - Telephone delivered care	- Haemoglobin A1c level - BP - Cholesterol level - Performance measure - Behavioural adherence (medication) - Protocol adherence	The ‘medical home’ is a team approach which may involve nurse- managed protocols. Nurse- managed protocols were associated with: - A highly variable mean decrease in HbA1c level. - A mean decrease in systolic and diastolic BP. - A mean decrease in total and low- density lipoprotein (LDL) cholesterol levels. Nurse- managed protocols were statistically significantly more likely to achieve target total cholesterol levels than control protocols. Effects of lifestyle changes and medication adherence showed an overall pattern of small positive effects associated with nurse- managed protocols. Adherence to protocol: pharmacologic therapy was started or doses were increased by nurses following treatment protocols more often than in usual care groups. Team approaches using nurse- managed protocols help improve health outcomes among patients with moderately severe diabetes, hypertension and hyperlipidemia.	22
9. Smith et al., England, 2014	To review the current literature on the participation and roles of APRNs/ Physician assistants (PAs) in providing cancer screening and prevention recommendations in primary care settings in the USA.	Systematic review. 15 quantitative studies included: - 3 intervention studies - 12 observational studies	- Adults (Smoking) - Pregnant women - Primary care settings - Private practices - Primary care health centres - Study hospitals - Obstetric clinic - Hospital ambulatory settings	7 studies reported outcomes on screening for - Cervical cancer (Pap test) - Breast cancer (Mammogram) - Colorectal cancer 10 studies reported outcomes on cancer prevention recommendations for - smoking cessation - diet - physical activity	Cervical cancer screening: - Physicians who work in teams that include NPs and PAs are more supportive of NPs and PAs performing Pap tests than physicians who do not practice in provider teams that include NPs and PAs. - The annual rate of women screened for cervical cancer by a NP increased significantly at the intervention location. Breast cancer screening: - 69- 91% of the patients who see NPs receive mammograms. - NPs recommend a similar number of mammograms as physicians. - The annual rate of mammography screening increased more among women seen at the NP screening recommendation site. Colorectal cancer screening: - Findings about APRN/PAs involvement in colorectal screening are mixed. - 2 studies showed physicians reporting more colorectal cancer screening than APRN/PAs. Smoking cessation recommendations: - Both physicians and APRN/PAs report frequently providing smoking cessation recommendations. - Patients are more likely to receive recommendations for smoking cessation during visits with NPs than during visits without NPs. Diet and physical activity recommendations: APRN/PAs do not frequently provide recommendations on diet and physical activity (12–52%), they do provide more recommendations than their physician counterparts (3–15%).	13
10. Snaterse et al., The Netherlands, 2016	To systematically review the available evidence on the efficacy of nurse- coordinated care (NCC) in secondary prevention of coronary heart diseases.	Systematic review and meta- analysis. 18 quantitative studies included: - 18 randomized controlled trials	- Patients with coronary heart diseases (adults) - Hospital setting - Outpatient clinics - Community health clinic - Secondary prevention unit - General practices	30 NCC outcomes were measured. Observed outcomes were grouped into four categories: - Risk factor levels - Clinical events - Patient perceived health - Guideline adherence	NCC programs were grouped into three strategies: - Risk factor management: education, lipid/BP control, advice on healthy diet and encouraging physical activity, prescription and or titration of drug therapy, enhancing adherence and smoking cessation counselling. - Multidisciplinary consultation: involvement of multidisciplinary team (>2 disciplines), consultation with general practitioner, referral to specialized disciplines. - Shared decision- making: personalized action plan, goal setting for cardiac risk factor control and family support. Effective components regarding behavioural interventions were goal setting for cardiac risk factor control plus identification of barriers. 8 Trials found positive outcomes for NCC compared with usual care: - Risk factor levels: total cholesterol, LDL cholesterol, triglyceride, pharmacological treatment, BP, diet, SCORE (a comprehensive cardiovascular risk algorithm designed for the primary prevention setting) and smoking cessation. - Clinical events: all- cause and cardiovascular readmission (days). - Guideline adherence.	21
11. Stalpers et al., The Netherlands, 2015	To systematically review the literature and to provide an overview of associations between characteristics of the nurse work environment (e.g., nurse staffing, nurse- physician collaboration) and five nurse- sensitive patient outcomes (i.e., delirium, malnutrition, pain, patient falls and pressure ulcers).	Systematic review. 29 quantitative studies included: - 1 randomized controlled trial - 28 observational studies	- Hospitalized patients - Hospital setting: surgical/general/emergency/intensive care/obstetric/cardiology/cardiothoracic surgery/respiratory units.	Nurse- sensitive outcomes: - 12 studies examined pressure ulcers. - 11 studies examined patient falls. - 6 studies analysed both pressure ulcers and patient falls among which one also elaborated on pain management.	Patient falls: - Collaborative nurse- physician relationships: 2/3 studies reported significant associations. Specifically, positively appreciated communication was associated with fewer adverse events and lower number of patient falls. Pressure ulcers: - Collaborative nurse- physician relationships: positively appreciated communication was associated with a lower number of pressure ulcers. Another study did not find significant associations.	15

Four systematic reviews performed a meta-analysis [21, 26–28]. The methodological quality of the included review articles varies from moderate [20, 24] to high [21, 28, 29]. Nine review articles included studies that were conducted in both a primary care setting and a hospital setting [19–24, 26, 28, 29]. Two review articles included studies that were exclusively conducted in a hospital setting [25, 27].

The eleven systematic reviews included a total of 285 different primary studies, the number of primary studies included in the review articles varies from 6 to 69. Most of the primary studies were included only once in a review, with the exception of 12 papers that were included in two reviews. Additional file 2 presents a list of all primary studies included in at least one of the reviews.

Table 3 presents the main findings of the meta-analyses. Four different review articles are presented. The table includes: intervention, control group and the different outcomes. The number of studies within the systematic review and the total number of patients are presented, followed by the (weighted median) effect size, a measure of heterogeneity and an appraisal of the quality of evidence/risk of bias (if available). The included systematic reviews provided no information on the performance of a statistical process for small-study effects. The table shows that interdisciplinary teams targeting either informational or management continuity had a positive impact, with a weighted median effect size (95% confidence interval) of respectively 2.0% (−0.03, 3.20) and 2.0% (−1.90, 3.20), on the quality of life of patients diagnosed with cancer. A measure of heterogeneity was not available. The quality of evidence of the included research articles, according to GRADE, was rated very low. Team based models of end-of-life care (home and comprehensive) caused a decrease in the number of people admitted to hospital and an increase of the number of people dying at home. Nurse-coordinated care as well as nurse-managed protocols had a positive effect on patients’ blood pressure and caused a decrease in patients’ low-density lipoprotein cholesterol levels.

**Table 3:** Meta-analyses (*n* = 4)

Aubin et al., 2012							
Intervention	Control	Outcome	Number of studies	Number of patients	Median effect size^a^ % (95% BCI)^b^	Hetero-geneity	Quality of evidence: GRADE
Interdisciplinary teams (targeting informational continuity)	Usual care	Functional status	11	3057	0 (−3.40, 2.70)	NAV	Very low
		Physical status	16	3589	0 (−0.50, 0.50)	NAV	Very low
		Psychological status	13	3228	−0.24 (−3.00, 0.02)	NAV	Very low
		Social status	4	589	−0.01 (−10.70, 0.30)	NAV	Very low
		**Global quality of life**	**9**	**2472**	**2.0 (−0.03, 3.20)**	**NAV**	**Very low**
Interdisciplinary teams (targeting management continuity)	Usual care	Functional status	11	2612	0 (−3.40, 2.00)	NAV	Very low
		Physical status	18	3439	0 (−0.50, 0.03)	NAV	Very low
		Psychological status	15	3687	−1.1 (−6.30, 0.00)	NAV	Very low
		Social status	4	528	−0.7 (−7.00, 0.30)	NAV	Very low
		**Global quality of life**	**7**	**1717**	**2.0 (−1.90, 3.20)**	**NAV**	**Very low**
Health Quality Ontario, 2014							
Intervention	Control	Outcome	Number of studies	Number of patients	Effect size (95% CI)	Hetero-geneity	Quality of evidence: GRADE
Home team-based model of care	Medicare guidelines for home health care	**Home death (number of people)**	**1**	**310**	**Odds ratio 2.20 (1.30, 3.72)**	**NA**	Low
		**Hospital admission (number of people admitted to hospital)**	**1**	**310**	**Odds ratio 0.39 (0.24, 0.62)**	**NA**	Low
Home (indirect) team-based model of care	Usual care by a management care organization	**Advanced care planning (number of people)**	**1**	**190**	**Odds ratio 1.30 (0.58, 2.90)**	**NA**	Very low
Hospital team-based model of care	Hospital care/primary care team only	**Advanced care planning**	**2**	**616**	**Odds ratio** **2.77 (0.48, 16.11)**	**I-square** **48%**	Very low
Comprehensive team-based model of care	Usual care	**Home death (number of people)**	**1**	**434**	**Odds ratio** **1.89 (1.13, 3.16)**	**NA**	Moderate
		**Nursing home death (number of people)**	**1**	**434**	**Odds ratio 0.37 (0.20, 0.67)**	**NA**	Moderate
		**Hospital admission**	**1**	**434**	**Odds ratio 0.90 (0.42, 1.89)**	**NA**	Moderate
Comprehensive, early start, team-based model of care	Routine oncologic care	**Hospital admission**	**1**	**151**	**Odds ratio 0.84 (0.34, 2.03)**	**NA**	Low
Shaw et al., 2014							
Intervention	Control	Outcome	Number of studies	Number of patients	Effect size (95% CI)	Hetero-geneity	Quality of evidence- risk of bias
Nurse-managed protocols	Usual care	**Systolic blood pressure (difference in mmHg)**	**12**	**Intervention:5244** **Control:4980**	**Weighted mean difference − 3.68 (−6.31, −1.05)**	**I-square 75.1%**	According to the approach recommended by the Agency for Healthcare Research and Quality: 4 articles: Low risk of bias/good quality 12 articles: Moderate risk of bias/fair quality 2 articles: High risk of bias/poor quality
	Usual care	**Diastolic blood pressure (difference in mmHg)**	**12**	**Intervention:5244** **Control:4980**	**Weighted mean difference − 1.56 (−2.76, −0.36)**	**I-square 75.1%**	
	Usual care	**Total cholesterol (difference in mg/dL)**	**9**	**Intervention:1879** **Control:1615**	**Weighted mean difference − 9.37 (−20.77, 2.02)**	**I-square 90.8%**	
	Usual care	**Low-density lipoprotein cholesterol (difference in mg/dL)**	**6**	**Intervention:564** **Control:555**	**Weighted mean difference − 12.07 (−28.27, 4.13)**	**I-square 89.1%**	
	Usual care	**Hemoglobin A1c level**	**8**	**Intervention:1444** **Control: 1189**	**Weighted mean difference − 0.40 (−0.70, −0.10)%**	**I-square** **69.8%**	
Snaterse et al., 2016							
Intervention	Control	Outcome	Number of studies	Number of patients	Effect size (95% CI)	Hetero-geneity	Quality of evidence– risk of bias
Nurse-coordinated care	Usual care	**Blood pressure (difference in mmHg)**	**7**	**3514**	**Weighted mean difference** **−2.96 (−4.40, −1.53)**	**I-square 37.1%** ***p*** **= 0.146**	Cochrane Collaboration’s risk of bias tool: low/unclear risk of bias.
	Usual care	**Low-density lipoprotein cholesterol (difference in mmol/L)**	**8**	**3441**	**Weighted mean difference** **−0.23 (−0.36, −0.10)**	**I-square** **74.3%** ***p*** **= 0.000**	
	Usual care	**Smoking cessation rates (Relative risk of quitting)**	**8**	**3265**	**Relative risk** **1.25 (1.09, 1.43)%**	**I-square** **0.0%** ***p*** **= 0.459**	

Table 3 presents the results of the meta-analyses of four of the included systematic reviews. The different ‘collaboration interventions’ are presented, followed by the control group, patient outcomes, number of studies, number of patients, effect size, a measure of heterogeneity (if available) and a measure of quality of evidence/risk of bias (if available). The improved patient outcomes are written in bold

*NA* ‘not applicable’, *NAV* ‘not available’

^a^To handle the diverse set of outcomes within each individual study, the median value was computed of all the measured effects across all the outcomes of the same class. To pool the results from multiple studies, the median effect size was calculated for each class of outcome, by computing the median from all the median effects in outcomes obtained from individual studies. The researchers chose this pooling strategy to be consistent with the median approach used in other reviews [45–47]

^b^non-parametric bootstrap confidence intervals

Table 4 presents an overview of the systematic reviews that did not provide a meta-analysis. Seven different review articles are presented. The table includes: intervention, control group and the different outcomes. The number of studies within the systematic review and the total number of patients are presented, followed by a statement on heterogeneity (if available) and an appraisal of the quality of evidence/risk of bias (if available).

**Table 4:** Overview systematic reviews without meta- analysis (*n* = 7)

Intervention	Control	Outcome	Number of studies	Number of patients	Heterogeneity	Quality of evidence- risk of bias
Allen et al., 2014						
Discharge protocol and advanced practice nurse	Usual care	- Length of hospital stay - Length of time till re- hospitalization - Costs - Functional status - Depression - Patient satisfaction - Quality of life - GP satisfaction	5 (RCT)	918	Due to heterogeneity in the transitional care interventions and outcomes, data were not pooled.	Cochrane Collaboration’s tool – high risk of performance bias in the included research articles
General practitioner and primary care nurse models	Usual care		3 (RCT)	1949		
Health Quality Ontario, 2013						
Nurse and physician care	Physician care	- Hospitalizations - Length of stay - Mortality - ED visits - Specialist visits - Health- related quality of life - Patient satisfaction - Disease specific measures - Examination or medication prescribing - Health- system efficiencies - Number and length of primary health care visits - Physician workload	6 (RCT)	Intervention: 1403 Control:1538	Due to clinical heterogeneity in the study populations evaluated, and differences in provider roles and characteristics, the pooling of outcomes was thought to be inappropriate and a meta- analysis was not conducted.	Quality of evidence: GRADE Low- Moderate quality
Martin et al., 2010						
Inter- professional collaboration – new models of care	Usual care	- Mortality - Clinical outcomes - Functional outcomes - Social outcomes - Utilization of medical services - Patient- reported outcomes: quality of life, activities of daily living	14 (RCT)	Intervention: 2788 Control: 2563	NAV	NAV
Newhouse et al., 2011						
Nurse practitioner/clinical nurse specialist care groups	Care management exclusively by physicians	- Patient satisfaction - Self- reported perceived health - Functional status - Glucose control - Lipid control - Blood pressure - ED visits - Hospitalizations - Duration of mechanical ventilation - Length of stay - Mortality - Cost - Complications	69: 20 (RCT) + 49 (obser- vational)	NAV	Effect sizes were not calculated for the multiple outcomes. Because of the widely varying populations, definitions, time periods, and study designs. Also, the publications did not consistently include the necessary data to calculate effect size.	Quality assessment by the Jadad scale 46 articles: High quality 12 articles: Low quality
Renders et al., 2000						
Interventions targeted at health care professionals or the structure in which health care professionals deliver their care. A more enhanced nursing role.	Usual care	- Glycemic control - Micro- or macro- vascular complications - Cardiovascular risk factors - Hospital admissions - Mortality - Well- being - Perceived health - Quality of life - Functional status - Patient satisfaction	41: 27 (RCT) + 12 (CBA) + 2 (ITS)	48,598	Given the likely heterogeneity of interventions, there is decided a priori not to use meta- analysis to pool the results of studies. Differences in guidelines and also in methods and reference values to assess glycated hemoglobin meant that a uniform effect size could not be valued and presented, thereby hindering between- study comparisons.	The quality criteria applied to RCT’s, CBAs and ITS are described in detail in the EPOC module of the Cochrane library. Allocation concealment: 17 articles clearly concealed Blind outcome assessment: 20 articles adequate 16 articles partly adequate Reliable outcome assessment: 22 articles adequate
Smith et al., 2014						
Participation of APRNs/PAs in providing cancer screening and prevention recommendations in primary care settings	Cancer screening and prevention provider teams with physicians that do not include APRNs/PAs	- Cervical cancer (Pap test) - Breast cancer (Mammogram) - Colorectal cancer - Smoking cessation - Diet - Physical activity	15: 3 intervention studies +12 observational studies	NAV	NAV	NAV
Stalpers et al., 2015						
Nurse- physician collaboration	Usual care	- Pressure ulcers - Patient falls - Pain management	29: 1 RCT + 28 observational studies	NAV	Fundamental problems with assessing and comparing data from primary studies prevents conducting an adequate quantitative meta- analysis of the literature.	Dutch version of Cochrane’s critical appraisal instrument: validity: moderate reliability: moderate applicability: moderate

*NAV* ‘not available’, *RCT* ‘randomized controlled trial’, *CBA* ‘controlled before and after study’, *ITS* ‘interrupted time series’

Table 4 presents the ‘collaboration intervention’, control, patient outcome, number of studies, number of patients (if available), a statement on heterogeneity (if available) and a measure of quality of evidence/risk of bias (if available) of seven included systematic reviews that did not conduct a meta- analysis

All eleven articles describe the impact of collaboration between physicians and nurses on patient outcomes. Table 5 presents an overview of the different outcomes described in the review articles. Table 5 provides an overview of the improved patient outcomes (collaboration between physicians and nurses led to better results for these outcomes), Table 6 shows an overview of the equivalent patient outcomes (collaboration between physicians and nurses led to equal results for these outcomes) and Table 7 presents an overview of the mixed patient outcomes (collaboration between physicians and nurses led to better and/or equal and/or worse results for these outcomes). Blood pressure, patient satisfaction and hospitalization are the outcomes where three or more systematic reviews concluded better results when physicians and nurses collaborated, compared to usual care. Systematic reviews often described a combination of improved and equivalent patient outcomes when the included articles showed mixed results.

**Table 5:** Overview improved patient outcomes

Serum lipid levels	Newhouse et al., Snaterse et al.
Cervical and breast cancer screening	Smith et al.
Lower cost of care	Newhouse et al., Allen et al.
Patient falls	Stalpers et al.
Pressure ulcers	Stalpers et al.
Guideline adherence	Snaterse et al.
Total cholesterol	Health Quality Ontario 2013, Snaterse et al.
Low density lipoprotein cholesterol	Shaw et al., Snaterse et al.
Triglyceride	Snaterse et al.
Pharmacological treatment	Snaterse et al.
Blood pressure	Health Quality Ontario 2013, Shaw et al., Snaterse et al.
SCORE^a^	Snaterse et al.
All-cause and cardiovascular readmission days	Snaterse et al.
Reduction in HbA1c levels	Heath Quality Ontario 2013, Shaw et al.
Self-perceived health	Martin et al.
Life satisfaction	Aubin et al., Martin et al.
Symptom severity	Aubin et al., Health Quality Ontario 2014.
Quality of life	Aubin et al., Health Quality Ontario 2014.
Delay in re-hospitalization	Allen et al.
Improved referral to community services	Allen et al.
General practitioner satisfaction	Allen et al.
Discharge communication to general practitioners	Allen et al.
Informal caregiver satisfaction	Health Quality Ontario 2014.
Increase likelihood of dying at home (end-of-life care)	Health Quality Ontario 2014.
Decrease likelihood of dying in a nursing home (end-of-life care)	Health Quality Ontario 2014.
Reduction of intensive care unit admission	Health Quality Ontario 2014.
Number of clinical examinations for blood pressure, BMI^b^ and smoking status	Health Quality Ontario 2013.
Number of foot examinations (diabetes)	Health Quality Ontario 2013
Patient outcomes also presented in Tables 6 or 7	
Smoking cessation recommendations	Smith et al., Snaterse et al.
Hospital length of stay	Allen et al., Martin et al., Newhouse et al.
Diet	Snaterse et al.
Patient satisfaction	Aubin et al., Allen et al., Health Quality Ontario 2013, Health Quality Ontario 2014., Martin et al.
Hospitalization rates	Allen et al., Health Quality Ontario 2013, Health Quality Ontario 2014., Martin et al.
Emergency department visits	Health Quality Ontario 2014., Martin et al.
Glycaemic control	Renders et al.
Mortality	Martin et al.
Physical, emotional and social functioning	Martin et al.

Table 5 presents the patient outcomes that were found to be improved (by one or more systematic reviews) when physicians and nurses collaborate, compared to no collaboration

^a^ *SCORE* Systematic Coronary Risk Evaluation. It’s a comprehensive cardiovascular risk algorithm designed for the primary prevention setting

^b^ *BMI* Body Mass index

**Table 6:** Overview equivalent patient outcomes

Patient satisfaction	Allen et al., Newhouse et al.
Self-reported perceived health	Newhouse et al.
Functional status outcomes	Allen et al., Newhouse et al.
Glycaemic control	Newhouse et al.
Blood pressure control	Newhouse et al.
Emergency department visits	Newhouse et al.
Hospitalization rates	Health Quality Ontario 2014., Martin et al., Newhouse et al.
Mortality	Martin et al., Newhouse et al.
Hospital length of stay	Health Quality Ontario 2014., Martin et al., Newhouse et al.
Recommendation of mammograms	Smith et al.
Smoking cessation recommendations	Smith et al.
Diet and physical therapy recommendations	Smith et al.
Depression	Allen et al.
Number of clinical examination of cholesterol	Health Quality Ontario 2013.
Utilisation of medical services	Martin et al.
Number of transfers	Martin et al.
Physical, emotional and social functioning	Martin et al.
Activities of daily living (ADL)	Martin et al.

Table 6 presents the patient outcomes that were found to be equal (by one or more systematic reviews) when physicians and nurses collaborate, compared to no collaboration

**Table 7:** Overview mixed patient outcomes

Colorectal screening	Smith et al.
hospitalization rates	Allen et al.
Hospital length of stay	Allen et al.

Table 7 presents the patient outcomes that were found to be improved and/or equivalent and/or declined

Table 8 describes the collaboration between physicians and nurses in the different review articles. Collaboration was described as a ‘multidisciplinary’, ‘inter-disciplinary’ or ‘inter-professional’. Other health care providers are often part of the team [20, 21, 26, 27].

**Table 8:** Collaboration between physicians and nurses

Authors	Collaboration
1. Allen et al.	Collaboration between a general practitioner and a primary care nurse in transitional care. Collaboration between an advanced practice nurse and a physician during the discharge plan.
2. Aubin et al.	Interdisciplinary team models of care for patients with cancer. These interventions used organizational strategies such as staff organization and the creation of teams of healthcare professionals working together to care for patients. These interventions also used local consensus processes, formal integration of services, arrangement for follow- up, coordination of assessment and treatment, and implementation of follow- up care plans. The interdisciplinary treatment team included: medical oncology, social work, occupational therapy, nursing, nutrition and dietetics and pastoral care.
3. Health Quality Ontario 2013.	Nurses/nurse practitioners/registered nurses and physicians working in a partnership. Nurses who worked in this collaboration could have been substituting or supplementing aspects of physician care.
4. Health Quality Ontario 2014.	An end- of- life care team contained at least a medical doctor and a registered nurse. Other possible team members: - social worker - spiritual advisor - nutritionist - geriatrician - pharmacist - dietician Team services included: - symptom management - psychosocial care - development of patient care plans - end- of- life care planning - coordination of care
5. Martin et al.	Inter- professional collaboration in the care for elderly with (chronic) diseases. Collaboration between at least (advanced practice) nurses and (primary care) physicians. Other care providers: - social worker - physio- occupational therapist - pharmacist - psychiatrist - …
6. Newhouse et al.	Advanced practice registered nurses (APRN)/ clinical nurse specialists delivered care in collaboration with physicians.
7. Renders et al.	The multidisciplinary team was led by a nurse educator. There was a joint general practitioner- nurse review system in combination with arrangements for follow up.
8. Shaw et al.	Collaboration according to nurse- managed protocols in the care for adults with elevated cardiovascular risk.
9. Smith et al.	APRN and Practice Assistants (PAs) provided cancer screening and prevention recommendations in collaboration with physicians.
10. Snaterse et al.	Multidisciplinary consultation for patients with coronary heart diseases. Collaboration between nurses and general practitioners or cardiologists. The following strategies were used: - Risk factor management. - Multidisciplinary consultation. - Shared decision- making.
11. Stalpers et al.	Collaborative nurse- physician relationships in the care for hospitalized patients.

Table 8 presents an overview of the interpretation of collaboration between physicians and nurses (and other health care providers) within the eleven included systematic reviews

**Fig. 2:**
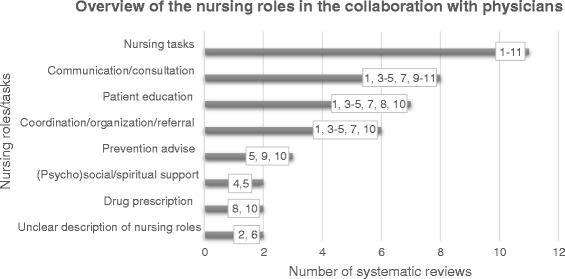
Overview of the nursing roles in the collaboration with physicians. Presents an overview of the 7 different nursing roles in collaboration with physicians within the eleven included systematic reviews. 2 systematic reviews failed to give a clear description of the nursing roles. The numbers within the graph represent the eleven included systematic reviews

Figure 2 presents the nursing roles/tasks in the collaboration with physicians in the included systematic reviews. The most frequently represented tasks are: specific nursing tasks (e.g. blood pressure control), communication/consultation tasks (e.g. communication with the multidisciplinary team), patient education tasks (e.g. lifestyle counseling) and coordination/organization/referral tasks (e.g. coordination of care, conducting a discharge planning). Two review articles did not clearly describe the tasks performed by the nurses.

## Discussion

Eleven systematic reviews describing the impact of collaboration between physicians and nurses on patient outcome were included in this overview of systematic reviews. Collaboration between physicians and nurses may have a positive impact on a number of patient outcomes and on a variety of pathologies.

Almost fifty different patient outcomes were described (Table 3). In most reviews, it was concluded that nurses do have added value. Maybe we observe some publication bias here since most of the author groups included nurses [30]. We also obtained mixed results in the other reviews. Blood pressure was the only patient outcome exclusively reported as improved in three different systematic reviews [19, 21, 28]. Two of them even performed a meta-analysis [21, 28]. Patient satisfaction is an improved patient outcome as well. No less than five different systematic reviews confirmed this [19, 20, 22, 26, 27]. However, two systematic reviews reported an equivalent patient satisfaction when physicians and nurses collaborated [22, 23]. Number of hospitalization is another improved patient outcome, confirmed by four different systematic reviews [19, 20, 22, 26]. However, three systematic reviews [20, 23, 26] also reported an equivalent number of hospitalizations and one [22] even reported an increase of hospitalizations when physicians and nurses collaborated. These mixed results make it difficult to make an accurate interpretation and conclusion towards the different patient outcomes.

Colorectal screening, hospital length of stay and health-related quality of life are three patient outcomes that also improved when physicians and nurses did not collaborate. However, only colorectal screening and health-related quality of life were merely categorized as negative outcomes. Allen et al. reported the length of hospital stay as a negative outcome. But the same review article also reported improvement in length of hospital stay, as well as two other review articles [20, 23]. Quality of life in general was reported as an improved outcome when physicians and nurses collaborated in two different review articles [26, 27].

The included systematic reviews often combined different interventions such as patient education [22, 29], medication adjustment [28], discharge planning protocol and shared decision making [21, 22] while measuring patient outcomes. Adding one or more interventions, besides collaboration between physicians and nurses, also makes it more difficult to determine which effect can be attributed to which intervention.

The evidence of collaboration between physicians and nurses on patient outcome can be applied to the primary care setting for almost all the measured patient outcomes. Only two systematic reviews included articles conducted in a hospital setting [25, 27]. Therefore, the improvement of global quality of life, and the decline of patient falls and pressure ulcers cannot be allotted to collaboration between physicians and nurses in the primary care setting.

### Collaboration

The different systematic reviews used a variety of terms describing the collaboration between health care providers including inter-professional collaboration, multidisciplinary collaboration, coordination, communication, teamwork and shared care. A clear definition and subsequent elaboration of the nature of the collaboration was lacking in most of the reviews. This is consistent with findings in the existing literature, where there seems to be no agreement on the use of terms to describe collaboration between health professionals [31]. This also makes it difficult to know how the collaboration translates itself in daily practice: were the studied collaborations between physicians and nurses merely focused on nurses performing dedicated tasks, based on physicians orders (a rather more instrumental collaboration)? Or were the studied collaborations focused on nurses’ competences and tasks with autonomous decision-making capacity, based on structured agreements between nurses and physicians (a rather more integrated collaboration)?

A total of 173 RCTs were finally included in this overview of systematic reviews. Although RCTs are the gold standard in establishing a firm evidence base in quantitative research, complex practice settings like health service settings, often require a more diverse methodology [32]. The relationship between teamwork and patient outcomes seems to be difficult to investigate with RCTs. A Cochrane review on interventions to promote collaboration between nurses and physicians concluded that rigorous evaluations are difficult to conduct. This is because the interventions are complex and the intermediate processes are difficult to assess [33]. Researchers in the United Kingdom increasingly use qualitative research methods alongside RCTs to gain a more comprehensive understanding of the impact of health service delivery [32]. Direct observation of collaborative practice in primary care settings holds promise as a method to better understand and articulate the complex phenomena of inter-professional collaboration. Despite methodological challenges, observation data may contribute in a unique way to the teamwork discourse by identifying elements of inter-professional collaboration that are not so obvious to caregivers when asked to self-report [34].

Open communication between physicians and nurses is an important element of collaboration that appeared to be appreciated [25]. More often, researchers reported that deficiencies in collaboration and communication between healthcare professionals have a negative impact on the provision of health care and patient outcomes [35–37]. In addition to open communication, trust, respect, shared leadership, recognition of unique contribution and collegiality are mentioned by researchers as enabling factors for good inter-professional relationships [38]. On the other hand, barriers to good inter-professional collaboration reported by researchers are time pressure, lack of explicit descriptions of each other’s roles and tasks (and therefore unawareness of one another’s roles and competencies), poor organizational support, absence of clear leadership, different standards and professional values, different aims and priorities, and vertical management structures with discriminatory power structures [39–41].

### Nursing roles

Although the review articles often lacked a comprehensive description of the nursing roles in collaboration with physicians, we identified seven different categories of nursing roles in the systematic reviews in our review. ‘Nursing tasks’ and ‘drug prescription’ may be more distinct instrumental roles or nursing tasks, and are probably based on physicians’ orders. ‘Communication/consultation’ and ‘coordination/organization/referral’ may be rather more related to integrated nursing roles with nurses’ autonomous decision-making capacity based on structured agreements between nurses and physicians. Existing literature confirms the nursing skill mix, and the shift from task delegation to team care with shared responsibilities [42, 43]. Two systematic reviews failed to describe the nature of the nursing roles and their responsibilities [23, 27]. Furthermore, nursing titles differed across the included systematic review articles and ranged from ‘advanced practice (registered) nurses’, ‘nurse practitioners’, ‘registered nurses’, ‘primary care nurses’, ‘clinical nurses specialists’ and ‘practice assistants’. The literature confirms the increasing diversity of the primary care workforce. A wider range of health professionals is included, such as those mentioned above [43]. The difference in professional titles might be attributed to a difference in education, which points out the importance of (postgraduate) education of nurses, especially in collaboration with physicians [29]. Expanding the role of primary care nurses is possible with appropriate training and on-going support from primary care physicians [44]. Improving care quality requires investing in a distinct primary care workforce that has followed a defined program of post-graduate training in primary care [2].

### Strengths and limitations

A comprehensive research was performed and the methodological quality of the included review articles was carefully assessed. Overall, the quality of available systematic reviews on this research topic appeared to be limited. 15 potentially useful systematic reviews were excluded based upon an inadequate methodological quality. The included systematic review articles were heterogeneous in terms of patient populations, setting, type of nurse and geographic region. Limited descriptions of the collaboration, and the different nursing and physician roles in the included systematic reviews are limitations of this overview of systematic reviews. The included systematic reviews often lacked a detailed description of the evidence of the different primary studies, therefore it is difficult to make conclusions about the strength of the evidence of the results. For future systematic reviews concerning this research topic we suggest to define more precisely the nature of the collaboration between the two professions and to provide a clear description of the concept of inter-professional collaboration.

Primary research articles concerning this research topic within the primary care setting are often limited to one pathology or diagnosis. However, the patient population in primary care presents itself with a wide range of pathologies. This overview of systematic reviews provides a more comprehensive view on the impact of collaboration between physicians and nurses in primary care on a wide variety of patient outcomes, for a wide range of patients.

Future research is necessary to define ‘integrated inter-professional collaboration’ in primary care more clearly, and to explore the impact of this collaboration on relevant patient and health care provider outcomes. These include hospital (re)admissions of patients with chronic conditions, patient satisfaction and primary health care provider satisfaction. We suggest using complementary methods to find a more robust evidence base for the collaboration of nurses and general practitioners in primary care.

### Implications for practice

This overview of systematic reviews provides a firm evidence base to engage practice nurses in general practices. Moreover, current and future challenges in primary care require a more integrated inter-professional collaboration instead of a task shift between general practitioners and nurses. Therefore, we recommend that collaboration between health care providers should be well described and discussed concerning roles, tasks and responsibilities of individual caretakers. A clear description is important in order to address the needs of the patient populations, and in order to address the individual patient needs.

## Conclusion

This overview of systematic reviews shows that collaboration between physicians and nurses may have a positive impact on a number of patient outcomes and on a variety of pathologies. To address future challenges of primary care, there is a need for more integrated inter-professional collaboration and sufficiently educated nurses.

## Additional files

Additional file 1: Search terms. Presents the search terms used within the different databases. (DOCX 12 kb)

Additional file 2: Reference list of primary research articles. Presents the references of all the primary research articles included in the systematic reviews. (DOCX 64 kb)

## Abbreviations

(HR)QOL: Health related quality of life
ADL: Activities of daily living
APRN: Advanced practice registered nurse
BCI: Bootstrap confidence interval
BMI: Body mass index
BP: Blood pressure
CAD: Coronary artery disease
CBA: Controlled before and after study
CHF: Chronic heart failure
CI: Confidence interval
CNS: Clinical nurse specialist
COPD: Chronic obstructive pulmonary disease
GP: General practitioner
ICU: Intensive care unit
ITS: Interrupted time series
LDL: Low density lipoprotein
NA: Not applicable
NAV: Not available
NCC: Nurse coordinated care
NICU: Neonatal intensive care unit
NP: Nurse practitioner
PA: Physician assistant
RCT: Randomized controlled trial
SCORE: Systematic coronary risk evaluation
