# Supplementary material for: An overview of systematic reviews on the collaboration between physicians and nurses and the impact on patient outcomes: what can we learn in primary care?
Source: BMC Fam Pract. 2017 Dec 22;18:110. doi: 10.1186/s12875-017-0698-x (PMC5741858; doi:10.1186/s12875-017-0698-x)
Supplement: Supplementary file 1 — Search terms. Presents the search terms used within the different databases. (DOCX 12 kb) [file 12875_2017_698_MOESM1_ESM.docx]

| **Database** | **Search terms** |
| --- | --- |
| MEDLINE | ((((((“general practice”[Mesh Terms]) OR “physicians”[Mesh Terms]) OR general pract*) OR doctor) OR physician) OR gp)  AND (((“nurses”[Mesh Terms]) OR “nursing”[Mesh Terms]) OR nurs*) AND  ((((((“patient care team”[Mesh Terms]) OR “cooperative behavior”[Mesh Terms]) OR collaborat*) OR team) OR teamwork) OR shared care) |
| COCHRANE | (((general practice) OR (physicians) OR (doctor) OR (GP)) AND ((nurses) OR (nursing)) AND ((patient care team) OR (cooperative behavior) OR (team) OR (teamwork) OR (shared care))) |
| EMBASE | 'general practice'/exp OR 'physician'/exp OR 'doctor'/exp AND ('nurse'/exp OR 'nursing'/exp OR nurs*) AND ('doctor nurse relation'/exp OR 'cooperation'/exp OR 'patient care'/exp OR (collaborative AND care)) NOT [22-5-2016]/sd AND [embase]/lim AND [review]/lim |
| CINAHL | (((general practice) OR (physicians) OR (doctor) OR (GP)) AND ((nurses) OR (nursing)) AND ((patient care team) OR (cooperative behavior) OR (team) OR (teamwork) OR (shared care))) |
