# Supplementary material for: An overview of systematic reviews on the collaboration between physicians and nurses and the impact on patient outcomes: what can we learn in primary care?
Source: BMC Fam Pract. 2017 Dec 22;18:110. doi: 10.1186/s12875-017-0698-x (PMC5741858; doi:10.1186/s12875-017-0698-x)
Supplement: Supplementary file 2 — Reference list of primary research articles. Presents the references of all the primary research articles included in the systematic reviews. (DOCX 64 kb) [file 12875_2017_698_MOESM2_ESM.docx]

**Reference list of primary research articles included in the systematic reviews.**

| References included primary studies. | 1.Allen et al. 2014 | 2.Aubin et al. 2012 | 3.Health Quality Ontario 2013 | 4.Health Quality Ontario 2014 | 5.Martin et al. 2010 | 6.Newhouse et al. 2011 | 7.Renders et al. 2000 | 8.Shaw et al. 2014 | 9.Smith et al. 2014 | 10.Snaterse et al. 2016 | 11.Stalpers et al. 2015 | Times cited |
| --- | --- | --- | --- | --- | --- | --- | --- | --- | --- | --- | --- | --- |
| Number of references included in systematic review. | 12 | 51 | 6 +2 | 12 | 14 | 69 | 41 | 18 +2 | 15 | 18 | 29 |  |
| Total number of references included in one or more systematic review: 285 + 4 |  |  |  |  |  |  |  |  |  |  |  |  |
| Addington-Hall JM, MacDonald LD, Anderson HR, Chamberlain J, Freeling P, Bland JM, et al. Randomised controlled trial of effects of coordinating care for terminally ill cancer patients. *BMJ* 1992;**305**(6865):1317–22. |  | x |  |  |  |  |  |  |  |  |  | 1 |
| Ahern, M., Imperial, J., & Lam, S. (2004). Impact of a designated hepatology nurse on the clinical course and quality  of life of patients treated with rebetron therapy for chronic hepatitis C. *Gastroenterology Nursing, 27*(4), 149-155. |  |  |  |  |  | x |  |  |  |  |  | 1 |
| Ahronheim J, Morrison S, Morris J, Baskin S, Meier D. Palliative care in advanced dementia: a randomized controlled trial and descriptive analysis. J Palliat Med. 2000;3(3):265-73. |  |  |  | x |  |  |  |  |  |  |  | 1 |
| Aigner, M.J., Drew, S., & Phipps, J. (2004). A comparative study of nursing home resident outcomes between care provided by nurse practitioners/physicians versus physicians only. *Journal* *of the American Medical Directors* *Association, 5*(1), 16-23. |  |  |  |  |  | x |  |  |  |  |  | 1 |
| Aiken LS, Butner J, Lockhart CA, Volk-Craft BE, Hamilton G, Williams FG. Outcome evaluation of a randomized trial of the PhoenixCare intervention: program of case management and coordinated care for the seriously chronically ill. J Palliat Med. 2006;9(1):111-26. |  |  |  | x |  |  |  |  |  |  |  | 1 |
| Aiken, L.H., Lake, E.T., Semaan, S.,Lehman, H.P., O’Hare, P.A., Cole, C.S., Frank, I. (1993). Nurse practitioner managed care for persons with HIV infection. *Journal of Nursing* *Scholarship, 25*(3), 172-177. |  |  |  |  |  | x |  |  |  |  |  | 1 |
| Allen JK. Coronary risk factor modification in women after coronary artery bypass surgery. Nurs Res 1996;45:260–5. |  |  |  |  |  |  |  |  |  | x |  | 1 |
| Allen JK, Blumenthal RS, Margolis S, et al. Nurse case management of hypercholesterolemia in patients with coronary heart disease: results of a randomized clinical trial. Am Heart J 2002;144:678–86. |  |  |  |  |  |  |  |  |  | x |  | 1 |
| Allen JK, Dennison-Himmelfarb CR, Szanton SL, et al. Community outreach and cardiovascular health (COACH) trial: a randomized, controlled trial of nurse practitioner/community health worker cardiovascular disease risk reduction in urban community health centers. Circ Cardiovasc Qual Outcomes 2011;4:595–602. |  |  |  |  |  |  |  |  |  | x |  | 1 |
| Allen KR, Hazelett S, Jarjoura D, Wickstrom GC, Hua K, Weinhardt J, et al. Effectiveness of a postdischarge care management model for stroke and transient ischemic attack: a randomized trial. J Stroke Cerebrovasc Dis. 2002;11(2):88-98. |  |  |  |  | x |  |  |  |  |  |  | 1 |
| Allison TG, Farkouh ME, Smars PA, et al. Management of coronary risk factors by registered nurses versus usual care in patients with unstable angina pectoris (a chest pain evaluation in the emergency room [CHEER] substudy). Am J Cardiol 2000;86:133–8. |  |  |  |  |  |  |  |  |  | x |  | 1 |
| Allison TG, Squires RW, Johnson BD, et al. Achieving National Cholesterol Education Program goals for low-density lipoprotein cholesterol in cardiac patients: importance of diet, exercise, weight control, and drug therapy. *Mayo Clin Proc*. 1999;74(5):466-73. |  |  |  |  |  |  |  | x |  |  |  | 1 |
| Aubert RE, Herman HW, Waters J, Moore W, Sutton D,  P1eterson BL. Nurse case management to improve glycemic  control in diabetic patients in a health maintenance  organization. A randomized, controlled trial.. *Annals of*  *Internal Medicine* 1998;**129**(8):605–12. |  |  |  |  |  |  | x | x |  |  |  | 2 |
| Bae, S.H., Mark, B., Fried, B., 2010a. Use of temporary nurses and nurse and patient safety outcomes in acute care hospital units. Health Care Manage. Rev. 35 (4), 333–344. |  |  |  |  |  |  |  |  |  |  | x | 1 |
| Bae, S.H., Mark, B., Fried, B., 2010b. Impact of nursing unit turnover on patient outcomes in hospitals. J. Nurs. Scholarsh. 42 (1), 40–49. |  |  |  |  |  |  |  |  |  |  | x | 1 |
| Baruffi, G., Strobino, D.M., & Paine, L.L. (1990). Investigation of institutional differences in primary cesarean birth rates. *Journal of Nurse-Midwifery,* *35*(5), 274-281. |  |  |  |  |  | x |  |  |  |  |  | 1 |
| Bauer MS, McBride L, Williford WO, Glick H, Kinosian B, Altshuler L, et al. Collaborative care for bipolar disorder: part I. Intervention and implementation in a randomized effectiveness trial. Psychiatr Serv. 2006;57(7):927-36. |  |  |  |  | x |  |  |  |  |  |  | 1 |
| Bauer MS, McBride L, Williford WO, Glick H, Kinosian B, Altshuler L, et al. Collaborative care for bipolar disorder: part II. Impact on clinical outcome, function, and costs. Psychiatr Serv. 2006;57(7):937-45. |  |  |  |  | x |  |  |  |  |  |  | 1 |
| Bebb C, Kendrick D, Coupland C, et al. A cluster randomised controlled trial of the effect of a treatment algorithm for hypertension in patients with type 2 diabetes. *Br J Gen Pract*.  2007;57(535):136-43. |  |  |  |  |  |  |  | x |  |  |  | 1 |
| Becker, D.M., Yanek, L.R., Johnson, W.R., Jr., Garrett, D., Moy, T.F., Reynolds, S.S., Becker, L.C. (2005). Impact of a community-based multiple risk factor intervention on cardiovascular risk in Black families with a history of premature coronary disease. *Circulation, 111*(10), 1298-1304. |  |  |  |  |  | x |  |  |  |  |  | 1 |
| Bellary S, O’Hare JP, Raymond NT, et al. Enhanced diabetes care to patients of south Asian ethnic origin (the United Kingdom Asian Diabetes Study): a cluster randomised controlled trial. *Lancet*. 2008;371(9626):1769-76. |  |  |  |  |  |  |  | x |  |  |  | 1 |
| Beney J, Devine EB, Chow V, Ignoffo RJ, Mitsunaga L,  Shahkarami M, et al. Effect of telephone follow-up on the  physical well-being dimension of quality of life in patients  with cancer. *Pharmacotherapy* 2002;**22**(10):1301–11. |  | x |  |  |  |  |  |  |  |  |  | 1 |
| Benjamin EM, Schneider MS, Hinchey KT. Implementing  practice guidelines for diabetes care using problembased  learning. A prospective controlled trial using firm  systems. *Diabetes Care* 1999;**22**(10):1672–8. |  |  |  |  |  |  | x |  |  |  |  | 1 |
| Bissinger, R.L., Allred, C.A., Arford, P.H., & Bellig, L.L. (1997). A cost-effectiveness analysis of neonatal nurse practitioners.  *Nursing Economic$, 15*(2), 92-99. |  |  |  |  |  | x |  |  |  |  |  | 1 |
| Blanchette, H. (1995). Comparison of obstetric outcome of a primary-care access clinic staffed by certified nurse-midwives and a private practice group of obstetricians in the same  community. *American Journal of Obstetrics and Gynecology, 172*(6), 1864-1871. |  |  |  |  |  | x |  |  |  |  |  | 1 |
| Bohnenkamp SK, McDonald P, Lopez AM, Krupinski E, Blackett A. Traditional versus telenursing outpatient management of patients with cancer with new ostomies. *Oncology Nursing Forum* 2004;**31**(5):1005–10. |  | x |  |  |  |  |  |  |  |  |  | 1 |
| Bonnema J, van Wersch AM, van Geel AN, Pruyn JF, Schmitz PI, PaulMA, et al. Medical and psychosocial effects of early discharge after surgery for breast cancer: randomized trial. *BMJ* 1998;**316**(7140):1267–71. |  | x |  |  |  |  |  |  |  |  |  | 1 |
| Borgmeyer, A., Gyr, P.M., Jamerson, P.A., & Henry, L.D. (2008). Evaluation of the role of the pediatric nurse practitioner in an inpatient asthma program. *Journal of Pediatric Health Care,* *22*(5), 273-281. |  |  |  |  |  | x |  |  |  |  |  | 1 |
| Boucher BJ, Claff HR, Edmonson M, Evans S, Harris  BTH, Hull AH. A pilot diabetic support service based on  family practice attenders: comparison with diabetic clinics  in east London. *Diabetic Medicine* 1987;**4**:480–4. |  |  |  |  |  |  | x |  |  |  |  | 1 |
| Boult C, Reider L, Frey K, Leff B, Boyd CM, Wolff JL, et al. Early effects of “Guided Care” on the quality of health care for multimorbid older persons: a cluster-randomized controlled trial. J Gerontol A Biol Sci Med Sci. 2008;63(3):321-7. |  |  |  |  | x |  |  |  |  |  |  | 1 |
| Boyes A, Newell S, Girgis A, McElduff P, Sanson-Fisher R. Does routine assessment and real-time feedback improve cancer patients’ psychosocial well-being?. *European Journal* *of Cancer Care* 2006;**15**(2):163–71. |  | x |  |  |  |  |  |  |  |  |  | 1 |
| Branger PJ, van’t Hooft A, van der Wouden JC, Moorman  PW, van Bemmel JH. Shared care for diabetes: supporting  communication between primary and secondary care.  *International Journal of Medical Informatics* 1999;**53**(2-3):  133–42. |  |  |  |  |  |  | x |  |  |  |  | 1 |
| Breckenridge-Sproat, S., Johantgen, M., Patrician, P., 2012. Influence of unit-level staffing on medication errors and falls in military hospitals. West. J. Nurs. Res. 34 (4), 455–474. |  |  |  |  |  |  |  |  |  |  | x | 1 |
| Brumley R, Enguidanos S, Jamison P, Seitz R, Morgenstern N, Saito S, et al. Increased satisfaction with care and lower costs: results of a randomized trial of in-home palliative care. J Am Geriatr Soc. 2007;55(7):993-1000. |  |  |  | x |  |  |  |  |  |  |  | 1 |
| Büla, C.J., Berod, A.C., Stuck, A.E., Alessi, C.A., Aronow, H.U., Santos-Eggimann, B., … Beck, J.C. (1999). Effectiveness of preventive in-home geriatric assessment in well functioning,  community-dwelling older people: Secondary analysis of a randomized trial. *Journal of the American* *Geriatrics Society, 47*(4), 389-395. |  |  |  |  |  | x |  |  |  |  |  | 1 |
| Burnes Bolton, L., Aydin, C.E., Donaldson, N., Brown, D.S., Sandhu, M., Fridman, M., Aronow, H.U., 2007. Mandated nurse staffing ratios in California: a comparison of staffing and nursing-sensitive outcomes pre-and post regulation. Policy Polit. Nurs. Pract. 8 (4), 238–250. |  |  |  |  |  |  |  |  |  |  | x | 1 |
| Butler, J., Abrams, B., Parker, J., Roberts, J.M., & Laros, R.K. (1993). Supportive nurse-midwife care is associated with a reduced incidence of cesarean section. *American Journal of Obstetrics* *and Gynecology, 168*(5), 1407-1413. |  |  |  |  |  | x |  |  |  |  |  | 1 |
| Callahan CM, Boustani MA, Unverzagt FW, Austrom MG, Damush TM, Perkins AJ, et al. Effectiveness of collaborative care for older adults with Alzheimer disease in primary care: a randomized controlled trial. JAMA. 2006;295(18):2148-57. |  |  |  |  | x | x |  |  |  |  |  | 2 |
| Campbell NC, Thain J, Deans HG, Ritchie LD, Rawles JM, Squair JL. Secondary prevention clinics for coronary heart disease: randomised trial of effect on health. BMJ. 1998;316(7142):1434-7. |  |  | x |  |  |  |  |  |  |  |  | 1 |
| Campbell NC, Ritchie LD, Thain J, et al. Secondary prevention in coronary heart disease: a randomised trial of nurse led clinics in primary care. Heart 1998;80:447–52. |  |  | x |  |  |  |  |  |  | x |  | 2 |
| Carlson A, Rosenqvist U. Diabetes care organization,  process, and patient outcomes: effects of a diabetes control  program. *Diabetes Educator* 1991;**17**(1):42–8. |  |  |  |  |  |  | x |  |  |  |  | 1 |
| Carlsson R, Lindberg G, Westin L, et al. Influence of coronary nursing management follow up on lifestyle after acute myocardial infarction. Heart 1997; 77:256–9. |  |  |  |  |  |  |  |  |  | x |  | 1 |
| Carrington MJ, Carrington MJ, Chan YK, et al. A multicenter, randomized trial of a nurse-led, home-based intervention for optimal secondary cardiac prevention suggests some benefits for men but not for women: the young at heart study.  Circ Cardiovasc Qual Outcomes 2013;6:379–89. |  |  |  |  |  |  |  |  |  | x |  | 1 |
| Chambliss, L.R., Daly, C., Medearis, A.L., Ames, M., Kayne, M., & Paul, R. (1992). The role of selection bias in comparing cesarean birth rates between physician and midwifery management. *Obstetrics & Gynecology,* *80*(2), 161-165. |  |  |  |  |  | x |  |  |  |  |  | 1 |
| Chang, Y.K., Hughes, L.C., Mark, B., 2006. Fitting in or standing out: nursing workgroup diversity and unit-level outcomes. Nurs. Res. 55 (6), 373– 380. |  |  |  |  |  |  |  |  |  |  | x | 1 |
| Cheung W, Aggarwal G, Fugaccia E, Thanakrishnan G, Milliss D, Anderson R, et al. Palliative care teams in the intensive care unit: a randomised, controlled, feasibility study. Crit Care Resusc. 2010;12(1):28-35. |  |  |  | x |  |  |  |  |  |  |  | 1 |
| Chew-Graham CA, Lovell K, Roberts C, Baldwin R, Morley M, Burns A, et al. A randomized controlled trial to test the feasibility of a collaborative care model for the management of depression in older people. Br J Gen Pract. 2007;57(538):364-70. |  |  |  |  | x |  |  |  |  |  |  | 1 |
| Coleman EA, Parry C, Chalmers S, Min SJ: The care transitions intervention: results of a randomized controlled trial. Arch Intern Med 2006, 166(17):1822–1828. | x |  |  |  |  |  |  |  |  |  |  | 1 |
| Counsell SR, Callahan CM, Clark DO, Tu W, Buttar AB, Stump TE, et al. Geriatric care management for low-income seniors: a randomized controlled trial. JAMA. 2007;298(22):2623-33. |  |  |  |  | x | x |  |  |  |  |  | 2 |
| Cragin, L. (2002). *Comparisons of care by nurse-midwives and obstetricians: Birth outcomes for moderate risk women.* Unpublished manuscript. University of California, San Francisco. |  |  |  |  |  | x |  |  |  |  |  | 1 |
| Cragin, L., & Kennedy, H.P. (2006). Linking obstetric and midwifery practice with optimal outcomes. *Journal of* *Obstetric, Gynecologic, & Neonatal* *Nursing, 35*(6), 779-785. |  |  |  |  |  | x |  |  |  |  |  | 1 |
| Dahle, K.L., Smith, J.S., Ingersoll, G.L., & Wilson, J.R. (1998). Impact of a nurse practitioner on the cost of managing  inpatients with heart failure. *The American Journal of Cardiology, 82*(5), 686-688, A8. |  |  |  |  |  | x |  |  |  |  |  | 1 |
| Davis, L.G., Riedmann, G.L., Sapiro, M., Minogue, J.P., & Kazer, R.R. (1994). Cesarean section rates in low-risk private patients managed by certified nurse-midwives and obstetricians. *Journal of Nurse-Midwifery, 39*(2), 91-97. |  |  |  |  |  | x |  |  |  |  |  | 1 |
| Day JL, Metcalfe J, Johnson P. Benefits provided by an  integrated education and clinical diabetes centre: a followup  study. *Diabet Med* 1992;**9**(9):855–9. |  |  |  |  |  |  | x |  |  |  |  | 1 |
| De Sonnaville JJ, Bouma M, Colly LP, Deville W, Wijkel  D, Heine RJ. Sustained good glycaemic control in NIDDM |  |  |  |  |  |  | x |  |  |  |  | 1 |
| DeBusk RF, Miller NH, Superko HR, et al. A case-management system for coronary risk factor modification after acute myocardial infarction. Ann Intern Med 1994;120:721–9. |  |  |  |  |  |  |  | x |  | x |  | 2 |
| Deeb LC, Pettijohn FP, Shirah JK, Freeman G. Interventions  among primary-care practitioners to improve care for  preventable complications of diabetes. *Diabetes Care* 1988;  **11**(3):275–80. |  |  |  |  |  |  | x |  |  |  |  | 1 |
| DeLano, C.F., Hirsh, L.D., & Schauberger, C.W. (1997). Nurse-midwifery at Gundersen clinic: A twenty year review. *Wisconsin Medical Journal,* *96*(6), 37-40. |  |  |  |  |  | x |  |  |  |  |  | 1 |
| Denver EA, Barnard M, Woolfson RG, et al. Management of uncontrolled hypertension in a nurse-led clinic compared with conventional care for patients with type 2 diabetes. *Diabetes Care*. 2003;26(8):2256-60. |  |  |  |  |  |  |  | x |  |  |  | 1 |
| de Wit R, van Dam F. From hospital to home care: a randomized controlled trial of a Pain Education Programme for cancer patients with chronic pain. *Journal of Advanced*  *Nursing* 2001;**36**(6):742–54. |  | x |  |  |  |  |  |  |  |  |  | 1 |
| Donaldson, N., Bolton, L.B., Aydin, C., Brown, D., Elashoff, J.D., Sandhu, M., 2005. Impact of California’s licensed nurse-patient ratios on unitlevel nurse staffing and patient outcomes. Policy Polit. Nurs. Pract. 6 (3), 198–210. |  |  |  |  |  |  |  |  |  |  | x | 1 |
| Drury M, Yudkin P, Harcourt J, Fitzpatrick R, Jones L, Alcock C, et al. Patients with cancer holding their own records: a randomised controlled trial. *British Journal of* *General Practice* 2000;**50**(451):105–10. |  | x |  |  |  |  |  |  |  |  |  | 1 |
| Du Pen SL, Du Pen AR, Polissar N, Hansberry J, Kraybill BM, Stillman M, et al. Implementing guidelines for cancer pain management: results of a randomized controlled clinical trial. *Journal of Clinical Oncology* 1999;**17**(1):361–70. |  | x |  |  |  |  |  |  |  |  |  | 1 |
| Enguidanos S, Gibbs N, Jamison P: From hospital to home: a brief nurse practitioner intervention for vulnerable older adults. Journal Of Gerontological Nursing 2012, 38(3):40–50. | x |  |  |  |  |  |  |  |  |  |  | 1 |
| Fanta, K., Cook, B., Falcone, R.A., Rickets, C., Schweer, L., Brown, R. L. Garcia, V.F. (2006). Pediatric trauma nurse practitioners provide excellent care with superior patient satisfaction for injured children. *Journal of* *Pediatric Surgery, 41*(1), 277-281. |  |  |  |  |  | x |  |  |  |  |  | 1 |
| Feder G, Griffiths C, Highton C, Eldridge S, Spence  M, Southgate L. Do clinical guidelines introduced with  practice based education improve care of asthmatic and  diabetic patients? A randomised controlled trial in general  practices in east London. *BMJ* 1995;**311**(7018):1473–8. |  |  |  |  |  |  | x |  |  |  |  | 1 |
| Fischer HH, Eisert SL, Everhart RM, et al. Nurse-run, telephone-based outreach to improve lipids in people with diabetes. *Am J Manag Care*. 2012;18(2):77-84. |  |  |  |  |  |  |  | x |  |  |  | 1 |
| Fischer H, Mackenzie T, McCullen K, et al. Design of a nurse-run, telephone-based intervention to improve lipids in diabetics. *Contemp Clin Trials*. 2008;29(5):809-16. |  |  |  |  |  |  |  | x |  |  |  | 1 |
| Fischler, N.R., & Harvey, S.M. (1995). Setting and provider of prenatal care: Association with pregnancy outcomes among low-income women. *Health* *Care for Women International, 16*(4), 309-321. |  |  |  |  |  | x |  |  |  |  |  | 1 |
| Frith, K.H., Anderson, E.F., Caspers, B., Tseng, F., Sanford, K., Hoyt, N.G., Moore, K., 2010. Effects of nurse staffing on hospital-acquired conditions and length of stay in community hospitals. Qual. Manag. Healthc. 19 (2), 147–155. |  |  |  |  |  |  |  |  |  |  | x | 1 |
| Gade G, Venohr I, Conner D, McGrady K, Beane J, Richardson RH, et al. Impact of an inpatient palliative care team: a randomized control trial. J Palliat Med. 2008;11(2):180-90. |  |  |  | x |  |  |  |  |  |  |  | 1 |
| Garrard, J., Kane, R.L., Radosevich, D.M., Skay, C.L., Arnold, S., Kepferle, L., Buchanan, J.L. (1990). Impact of geriatric nurse practitioners on nursinghome residents’ functional status, satisfaction, and discharge outcomes. *Medical Care, 28*(3), 271-283. |  |  |  |  |  | x |  |  |  |  |  | 1 |
| Gebauer C, Kwo CY, Haynes EF, Wewers ME: A nurse-managed smoking cessation intervention during pregnancy. J Obstet Gynecol Neonatal Nurs 1998, 27(1):47–53. |  |  |  |  |  |  |  |  | x |  |  | 1 |
| Giesler RB, Given B, Given CW, Rawl S, Monahan P, Burns D, et al. Improving the quality of life of patients with prostate carcinoma: a randomized trial testing the efficacy of a nurse-driven intervention. *Cancer* 2005;**104**(4):752–62. |  | x |  |  |  |  |  |  |  |  |  | 1 |
| Given B, Given CW, McCorkle R, Kozachik S, Cimprich B, Rahbar MH, et al. Pain and fatigue management: results of a nursing randomized clinical trial. Oncology Nursing Forum 2002; Vol. 29, issue 6:949–56. |  | x |  |  |  |  |  |  |  |  |  | 1 |
| Gomes B, Calanzani N, Curiale V, McCrone P, Higginson IJ. Effectiveness and cost-effectiveness of home palliative care services for adults with advanced illness and their caregivers. Cochrane Database of Systematic Reviews 2013;6(Art. No.:CD007760. DOI: 10.1002/14651858.CD007760.pub2.):1-279. |  |  |  | x |  |  |  |  |  |  |  | 1 |
| Goode, C.J., Blegen, M.A., Park, S.H., Vaughn, T., Spetz, J., 2011. Comparison of patient outcomes in magnet and non-magnet hospitals. J. Nurs. Adm. 41 (12), 517–523. |  |  |  |  |  |  |  |  |  |  | x | 1 |
| Goodwin JS, Satish S, Anderson ET, Nattinger AB, Freeman  JL. Effect of nurse case management on the treatment of older women with breast cancer. *Journal of the American* *Geriatrics Society* 2003;**51**(9):1252–9. |  | x |  |  |  |  |  |  |  |  |  | 1 |
| Gordon NF, English CD, Contractor AS, et al. Effectiveness of three models for comprehensive cardiovascular disease risk reduction. Am J Cardiol 2002;89:1263–8. |  |  |  |  |  |  |  |  |  | x |  | 1 |
| Gracias, V. H., Sicoutris, C. P., Stawicki, S.P., Meredith, D. M., Horan, A. D., Gupta, R. Schwab, C.W. (2008). Critical care nurse practitioners improve compliance with clinical practice guidelines in “semiclosed” surgical intensive care unit. *Journal of* *Nursing Care Quality, 23*(4), 338-344. |  |  |  |  |  | x |  |  |  |  |  | 1 |
| Grunfeld E, Fitzpatrick R, Mant D, Yudkin P, Adewuyi-Dalton R, Stewart J, et al. Comparison of breast cancer patient satisfaction with follow-up in primary care versus specialist care: results from a randomized controlled trial. *British Journal of General Practice* 1999;**49**(446):705–10. |  | x |  |  |  |  |  |  |  |  |  | 1 |
| Grunfeld E, Levine MN, Julian JA, Coyle D, Szechtman B, Mirsky D, et al. Randomized trial of long-term followup for early-stage breast cancer: a comparison of family physician versus specialist care. *Journal of Clinical Oncology* 2006;**24**(6):848–55. |  | x |  |  |  |  |  |  |  |  |  | 1 |
| Gunningberg, L., Donaldson, N., Aydin, C., Idyall, E., 2012. Exploring variation in pressure ulcer prevalence in Sweden and the USA: benchmarking in action. J. Eval. Clin. Pract. 18 (4), 904–910. |  |  |  |  |  |  |  |  |  |  | x | 1 |
| Halbert RJ, Leung KM, Nichol JM, Legorreta AP. Effect  of multiple patient reminders in improving diabetic  retinopathy screening. A randomized trial. *Diabetes Care*  1999;**22**(5):752–5. |  |  |  |  |  |  | x |  |  |  |  | 1 |
| Hanks GW, Robbins M, Sharp D, Forbes K, Done K, Peters  TJ, et al. The imPaCT study: a randomised controlled trial to evaluate a hospital palliative care team. *British Journal of* *Cancer* 2002;**87**(7):733–9. |  | x |  | x |  |  |  |  |  |  |  | 2 |
| Hansen FR, Poulsen H, Sørensen KH: A model of regular geriatric follow-up by home visits to selected patients discharged from a geriatric ward: a randomized controlled trial. Aging 1995, 7(3):202–206. | x |  |  |  |  |  |  |  |  |  |  | 1 |
| Hartmann P, GrusserM, Jorgens V. Structured public health  quality circle on the topic of diabetes management in general  practice [Strukturierte kassenärztliche Qualitätszirkel zum  Thema Diabetikerbetreuung in der Praxis [German]].  *Zeitschrift fur Fortbildung* 1995;**89**(4):415–8. |  |  |  |  |  |  | x |  |  |  |  | 1 |
| Hawkins DW. Clinical pharmacy functions in ambulatory  patient care. *Journal of Clinical Pharmacology* 1981;**21**(5-6):  245–50. |  |  |  |  |  |  | x |  |  |  |  | 1 |
| Heins, H.C., Nance, N.W., McCarthy, B.J., & Efird, C.M. (1990). A randomized trial of nurse-midwifery prenatal care to reduce low birth weight. *Obstetrics &* *Gynecology, 75*(3), 341-345. |  |  |  |  |  | x |  |  |  |  |  | 1 |
| Higginson IJ, Finlay IG, Goodwin DM, Hood K, Edwards AG, Cook A, et al. Is there evidence that palliative care teams alter end-of-life experiences of patients and their caregivers? J Pain Symptom Manage. 2003;25(2):150-68. |  |  |  | x |  |  |  |  |  |  |  | 1 |
| Hoffman, L.A., Tasota, F.J., Zullo, T.G., Scharfenberg, C., & Donahoe, M.P. (2005). Outcomes of care managed by an acute care nurse practitioner/attending physician team in a subacute medical intensive care unit. *American Journal of Critical Care,* *14*(2), 121-130. |  |  |  |  |  | x |  |  |  |  |  | 1 |
| Hopkins SC, Lenz ER, Pontes NM, Lin SX, Mundinger MO: Context of care or provider training: the impact on preventive screening practices. Prev Med 2005, 40(6):718–724. |  |  |  |  |  |  |  |  | x |  |  | 1 |
| Hoskins PL, Fowler PM, Constantino M, Forrest J, Yue  DK, Turtle JR. Sharing the care of diabetic patients between hospital and general practitioners: does it work?. *Diabetic*  *Medicine* 1993;**10**(1):81–6. |  |  |  |  |  |  | x |  |  |  |  | 1 |
| Hueston, W.J., & Rudy, M. (1993). A comparison of labor and delivery management between nurse midwives and family physicians. *The Journal of* *Family Practice, 37*(5), 449-454. |  |  |  |  |  | x |  |  |  |  |  | 1 |
| Houweling ST, Kleefstra N, van Hateren KJ, et al. Diabetes specialist nurse as main care provider for patients with type 2 diabetes. *Neth J Med*. 2009;67(7):279-84. |  |  |  |  |  |  |  | x |  |  |  | 1 |
| Houweling ST, Kleefstra N, van Hateren KJ, et al. Can diabetes management be safely transferred to practice nurses in a primary care setting? A randomised controlled trial. *J* *Clin Nurs*. 2011;20(9-10):1264-72. |  |  | x |  |  |  |  | x |  |  |  | 2 |
| Hughes SL, Cummings J, Weaver F, Manheim L, Braun B, Conrad K. A randomized trial of the cost effectiveness of VA hospital-based home care for the terminally ill. *Health* *Service Research* 1992;**26**(6):801–17. |  | x |  |  |  |  |  |  |  |  |  | 1 |
| Hurwitz B, Goodman C, Yudkin J. Prompting the clinical  care of non-insulin dependent (type II) diabetic patients in  an inner city area: one model of community care. *BMJ*  1993;**306**(6878):624–30. |  |  |  |  |  |  | x |  |  |  |  | 1 |
| Inglis SC, Pearson S, Treen S, Gallasch T, Horowitz JD, Stewart S. Extending the horizon in chronic heart failure: effects of multidisciplinary, home-based intervention relative to usual care. Circulation. 2006;114(23):2466-73. |  |  |  |  | x |  |  |  |  |  |  | 1 |
| Jaber LA, Halapy H, Fernet M, Tummalapalli S, Diwakaran  H. Evaluation of a pharmaceutical care model on diabetes  management. *Annals of Pharmacotherapy* 1996;**30**(3):  238–43. |  |  |  |  |  |  | x |  |  |  |  | 1 |
| Jackson, D.J., Lang, J.M., Ecker, J., Swartz, W.H., & Heeren, T. (2003). Impact of collaborative management and early admission in labor on method of delivery. *Journal of Obstetric,*  *Gynecologic, & Neonatal Nursing, 32*(2), 147-157. |  |  |  |  |  | x |  |  |  |  |  | 1 |
| Jackson, D.J., Lang, J.M., Swartz, W.H., Ganiats, T.G., Fullerton, J., Ecker, J., & Nguyen, U. (2003). Outcomes, safety, and resource utilization in a collaborative care birth center program compared with traditional physician based perinatal care. *American* *Journal of Public Health, 93*(6), 999-1006. |  |  |  |  |  | x |  |  |  |  |  | 1 |
| Jefford M, Baravelli C, Dudgeon P, Dabscheck A, Evans M, Moloney M, et al. Tailored chemotherapy information faxed to general practitioners improves confidence in managing adverse effects and satisfaction with shared care: results from a randomized controlled trial. *Journal of* *Clinical Oncology* 2008;**26**(14):2272–7. |  | x |  |  |  |  |  |  |  |  |  | 1 |
| Jiang, H.J., Stocks, C., Wong, C.J., 2006. Disparities between two common data sources on hospital nurse staffing. J. Nurs. Scholarsh. 38 (2), 187– 193. |  |  |  |  |  |  |  |  |  |  | x | 1 |
| Jiang X, Sit JW, Wong TK. A nurse-led cardiac rehabilitation programme improves health behaviours and cardiac physiological risk parameters: evidence from Chengdu, china. J Clin Nurs 2007;16:1886–97. |  |  |  |  |  |  |  |  |  | x |  | 1 |
| Johansson B, Berglund G, Glimelius B, Holmberg L, Sjoden PO. Intensified primary cancer care: a randomized study of home care nurse contacts. *Journal of Advanced Nursing* 1999;**30**(5):1137–46. |  | x |  |  |  |  |  |  |  |  |  | 1 |
| Jolly K, Bradley F, Sharp S, et al. Randomised controlled trial of follow up care in general practice of patients with myocardial infarction and angina: final results of the Southampton heart integrated care project (SHIP). The SHIP Collaborative Group. BMJ 1999;318:706–11. |  |  |  |  |  |  |  |  |  | x |  | 1 |
| Jordhoy MS, Fayers P, Loge JH, Ahlner-Elmqvist M, Kaasa S. Quality of life in palliative cancer care: Results from a cluster randomized trial. *Journal of Clinical Oncology* 2001; **19**(18):3884–94. |  | x |  |  |  |  |  |  |  |  |  | 1 |
| Jordhoy MS, Fayers P, Saltnes T, Ahlner-Elmqvist M, Jannert M, Kaasa S. A palliative-care intervention and death at home: a cluster randomised trial. Lancet. 2000;356(9233):888-93. |  |  |  | x |  |  |  |  |  |  |  | 1 |
| Jorstad HT, von Birgelen C, Alings AM, et al. Effect of a nurse-coordinated prevention programme on cardiovascular risk after an acute coronary syndrome: main results of the RESPONSE randomised trial. Heart 2013;99:1421–30. |  |  |  |  |  |  |  |  |  | x |  | 1 |
| Kane, R.L., Flood, S., Bershadsky, B., & Keckhafer, G. (2004). Effect of an innovative Medicare managed careprogram on the quality of care for nursing home residents. *Gerontologist,* 44(1), 95-103. |  |  |  |  |  | x |  |  |  |  |  | 1 |
| Kane RL, Wales J, Bernstein L, Leibowitz A, Kaplan S. A randomised controlled trial of hospice care. *Lancet* 1984;**1**  (8382):890–4. |  | x |  |  |  |  |  |  |  |  |  | 1 |
| Karlowicz, M.G., & McMurray, J.L. (2000). Comparison of neonatal nurse practitioners’ and pediatric residents care of extremely low-birth-weight infants. *Archives of Pediatrics and Adolescent* *Medicine, 154*(11), 1123-1126. |  |  |  |  |  | x |  |  |  |  |  | 1 |
| Kendall-Gallagher, D., Blegen, M.A., 2009. Competence and certification of registered nurses and safety of patients in intensive care units. Am. J. Crit. Care 18 (2), 106–113. |  |  |  |  |  |  |  |  |  |  | x | 1 |
| Khunti K, Stone M, Paul S, et al. Disease management programme for secondary prevention of coronary heart disease and heart failure in primary care: a cluster  randomised controlled trial. Heart 2007;93:1398–405. |  |  | x |  |  |  |  |  |  | x |  | 2 |
| King M, Jones L, McCarthy O, Rogers M, Richardson A, Williams R, et al. Development and pilot evaluation of a  complex intervention to improve experienced continuity of  care in patients with cancer. *British Journal of Cancer* 2009;  **100**(2):274–80. |  | x |  |  |  |  |  |  |  |  |  | 1 |
| Kinmonth AL,Woodcock A, Griffin S, Spiegal N, Campbell  MJ. Randomised controlled trial of patient centred care of  diabetes in general practice: impact on current well being  and future disease risk. The Diabetes Care From Diagnosis  Research Team. *BMJ* 1998;**317**(7167):1202–8. |  |  |  |  |  |  | x |  |  |  |  | 1 |
| Koinberg IL, Fridlund B, Engholm GB, Holmberg L. Nurse-led follow-up on demand or by a physician after breast cancer surgery: a randomised study. *European Journal* *of Oncological Nursing* 2004;**8**(2):109-17; discussion 118-20. |  | x |  |  |  |  |  |  |  |  |  | 1 |
| Kousgaard KR, Nielsen JD, Olesen F, Jensen AB. General practitioner assessment of structured oncological information accompanying newly referred cancer patients. *Scandinavian Journal of Primary Health Care* 2003;**21**(2):110–4. |  | x |  |  |  |  |  |  |  |  |  | 1 |
| Krapohl, G., Manojlovich, M., Redman, R., Zhang, L., 2010. Nursing speciality certification and nursing-sensitive patient outcomes in the intensive care unit. Am. J. Crit. Care 19 (6), 490–498. |  |  |  |  |  |  |  |  |  |  | x | 1 |
| Kravitz RL, Delafield JP, Hays RD, Drazin R, Conolly M. Bedside charting of pain levels in hospitalized patients with cancer: a randomized controlled trial. *Journal of Pain and* *Symptom Management* 1996;**11**(2):81–7. |  | x |  |  |  |  |  |  |  |  |  | 1 |
| Krein SL, Klamerus ML, Vijan S, Lee JL, Fitzgerald JT, Pawlow A, et al. Case management for patients with poorly controlled diabetes: a randomized trial. Am J Med. 2004;116(11):732-9. |  |  |  |  | x |  |  |  |  |  |  | 1 |
| Krichbaum, K. (2007). GAPRN postacute care coordination improves hip fracture outcomes. *Western Journal of* *Nursing Research, 29*(5), 523-544. |  |  |  |  |  | x |  |  |  |  |  | 1 |
| Kutzleb, J., & Reiner, D. (2006). The impact of nurse-directed patient education on quality of life and functional capacity in people with heart failure. *Journal of the American Academy of*  *Nurse Practitioners, 18*(3), 116-123. |  |  |  |  |  | x |  |  |  |  |  | 1 |
| Lambing, A.Y., Adams, D.L., Fox, D.H., & Divine, G. (2004). Nurse practitioners’ and physicians’ care activities and clinical outcomes with an inpatient geriatric population. *Journal of the* *American Academy of Nurse* *Practitioners, 16*(8), 343-352. |  |  |  |  |  | x |  |  |  |  |  | 1 |
| Laurant MGH, Hermens RPMG, Braspenning JCC, Sibbald B, Grol RPTM. Impact of nurse practitioners on workload of general practitioners: randomised controlled trial. BMJ. 2004;328(7445):927-30. |  |  | x |  |  |  |  |  |  |  |  | 1 |
| Legorreta AP, Peters AL, Ossorio C, Lopez RJ, Jatulis D,  Davidson MB. Effect of a comprehensive nurse-managed  diabetes program: an HMO prospective study. *Am J Man*  *Care* 1996;**2**:1024–30. |  |  |  |  |  |  | x |  |  |  |  | 1 |
| Legrain S, Tubach F, Bonnet-Zamponi D, Lemaire A, Aquino J-P, Paillaud E, Taillandier-Heriche E, Thomas C, Verny M, Pasquet B, Moutet Aline L, Lieberherr D, Lacaille S: A new multimodal geriatric discharge-planning intervention to  prevent emergency visits and rehospitalizations of older adults: The Optimization of Medication in AGEd multicenter randomized controlled trial. J Am Geriatr Soc 2011, 59(11):2017–2028. | x |  |  |  |  |  |  |  |  |  |  | 1 |
| Lenaway, D., Koepsell, T.D., Vaughan, T., Van Belle, G., Shy, K., & Cruz-Uribe, F. (1998). Evaluation of a public-private certified nurse-midwife maternity program for indigent women.  *American Journal of Public Health, 88*(4), 675-679. |  |  |  |  |  | x |  |  |  |  |  | 1 |
| Lenz, E.R., Mundinger, M.O., Hopkins, S.C., Lin, S.X., & Smolowitz, J.L. (2002). Diabetes care processes and outcomes in patients treated by nurse practitioners or physicians. *The*  *Diabetes Educator, 28*(4), 590-598. |  |  | x |  |  | x |  |  |  |  |  | 2 |
| Lenz, E.R., Mundinger, M.O., Kane, R.L., Hopkins, S.C., & Lin, S.X. (2004). Primary care outcomes in patients treated by nurse practitioners or physicians: Two-year follow-up. *Medical Care Research and Review,* *61*(3), 332-351. |  |  |  |  |  | x |  |  |  |  |  | 1 |
| Lim W, Lambert S, Gray L: Effectiveness of case management and post-acute services in older people after discharge. Med J Aust 2003, 178(6):262–266. | x |  |  |  |  |  |  |  |  |  |  | 1 |
| Lin SX, Gebbie KM, Fullilove RE, Arons RR: Do nurse practitioners make a difference in provision of health counseling in hospital outpatient departments? J Am Acad Nurse Pract 2004, 16(10):462–466. |  |  |  |  |  |  |  |  | x |  |  | 1 |
| Litaker, D., Mion, L.C., Planavsky, L., Kippes, C., Mehta, N., & Frolkis, J. (2003). Physician-nurse practitioner teams in chronic disease management: The impact on costs, clinical effectiveness, and patients’ perception of care. *Journal of Interprofessional Care, 17*(3), 223-237. |  |  | x |  |  | x |  |  |  |  |  | 2 |
| Litzelman DK, Slemenda CW, Langefeld CD, Hayes LM,  Welch MA, Bild DE. Reduction of lower extremity clinical  abnormalities in patients with non-insulin-dependent  diabetes mellitus. A randomized, controlled trial. *Annals*  *of Internal Medicine* 1993;**119**(1):36–41. |  |  |  |  |  |  | x |  |  |  |  | 1 |
| Liu LN, Li CY, Tang ST, Huang CS, Chiou AF. Role of continuing supportive cares in increasing social support and reducing perceived uncertainty among women with newly diagnosed breast cancer in Taiwan. *Cancer Nursing* 2006;**29**(4):273–82. |  | x |  |  |  |  |  |  |  |  |  | 1 |
| Lobach DF, Hammond WE. Computerized decision  support based on a clinical practice guideline improves  compliance with care standards. *American Journal of*  *Medicine* 1997;**102**(1):89–98. |  |  |  |  |  |  | x |  |  |  |  | 1 |
| Low, L.K., Seng, J.S., Murtland, T.L., & Oakley, D. (2000). Clinician-specific episiotomy rates: Impact on perineal outcomes. *Journal of Midwifery &* *Women’s Health, 45*(2), 87-93. |  |  |  |  |  | x |  |  |  |  |  | 1 |
| Luker K, Beaver K, Austin L, Leinster SJ. An evaluation of  information cards as a means of improving communication  between hospital and primary care for women with breast  cancer. *Journal of Advanced Nursing* 2000;**31**(5):1174–82. |  | x |  |  |  |  |  |  |  |  |  | 1 |
| MacDorman, M.F., & Singh, G.K. (1998). Midwifery care, social and medical risk factors, and birth outcomes in the USA. *British Medical Journal, 52*(5), 310-317. |  |  |  |  |  | x |  |  |  |  |  | 1 |
| MacMahon Tone J, Agha A, Sherlock M, et al. An intensive nurse-led, multiinterventional clinic is more successful in achieving vascular risk reduction targets than  standard diabetes care. *Ir J Med Sci*. 2009;178(2):179-86. |  |  |  |  |  |  |  | x |  |  |  | 1 |
| Mallidou, A.A., Cummings, G.G., Estabrooks, C.A., Giovannetti, P.B., 2011. Nurse speciality subcultures and patient outcomes in acute care hospitals: a multiple-group structural equation modeling. Int. J. Nurs. Stud. 48 (1), 81–93. |  |  |  |  |  |  |  |  |  |  | x | 1 |
| Mandelblatt J, Traxler M, Lakin P, Thomas L, Chauhan P, Matseoane S, Kanetsky P: A nurse practitioner intervention to increase breast and cervical cancer screening for poor, elderly black women. The Harlem study team. J Gen Intern Med 1993, 8(4):173–178. |  |  |  |  |  |  |  |  | x |  |  | 1 |
| Manojlovich, M., Antonakos, C.L., Ronis, D.L., 2009. Intensive care units, communication between nurses and physicians, and patients’ outcomes. Am. J. Crit. Care 18 (1), 21–30. |  |  |  |  |  |  |  |  |  |  | x | 1 |
| Manojlovich, M., Sidani, S., Covell, C.L., Antonakos, C.L., 2011. Nurse dose: linking staffing variables to adverse patient outcomes. Nurs. Res. 60 (4), 214–220. |  |  |  |  |  |  |  |  |  |  | x | 1 |
| Mark, B.A., Harless, D.W., McCue, M., Xu, Y., 2004. A longitudinal examination of hospital registered nurse staffing and quality of care. Health Serv. Res. 39 (2), 279–300. |  |  |  |  |  |  |  |  |  |  | x | 1 |
| Marrero DG, Vandagriff JL, Kronz K, Fineberg NS, Golden  MP, Gray D. Using telecommunication technology to  manage children with diabetes: the Computer-Linked  Outpatient Clinic (CLOC) Study. *Diabetes Educator* 1995;  **21**(4):313–9. |  |  |  |  |  |  | x |  |  |  |  | 1 |
| Mason JM, Freemantle N, Gibson JM, et al. Specialist nurse-led clinics to improve control of hypertension and hyperlipidemia in diabetes: economic analysis of the SPLINT  trial. *Diabetes Care*. 2005;28(1):40-6. |  |  |  |  |  |  |  | x |  |  |  | 1 |
| Mazze RS, Etzwiler DD, Strock E, Peterson K, McClave  CR 2nd, Meszaros JF, et al.Staged diabetes management.  Toward an integrated model of diabetes care. *Diabetes Care*  1994;**17 Suppl 1**:56–66. |  |  |  |  |  |  | x |  |  |  |  | 1 |
| Mazzuca SA, Vinicor F, Einterz RM, Tierney WM, Norton  JA, Kalasinski LA. Effects of the clinical environment on  physicians’ response to postgraduate medical education. *Am*  *Educ Research J* 1990;**27**:473–88. |  |  |  |  |  |  | x |  |  |  |  | 1 |
| McArdle JMC, George WD, McArdle CS, Smith DC, Moodie AR, Hughson AVM, et al. Psychological support for patients undergoing breast cancer surgery: a randomized study. *BMJ* 1996;**312**(7034):813–6. |  | x |  |  |  |  |  |  |  |  |  | 1 |
| McCloskey, B.A., Diers, D.K., 2005. Effects of New Zealand’s health reengineering on nursing and patient outcomes. Med. Care 43 (11), 1140–1146. |  |  |  |  |  |  |  |  |  |  | x | 1 |
| McCorkle R, Benoliel JQ, Donaldson G, Georgiadou F, Moinpour C, Goodell B. A randomized clinical trial of home nursing care for lung cancer patients 2155. *Cancer* 1989;**64**(6):1375–82. |  | x |  |  |  |  |  |  |  |  |  | 1 |
| McCorkle R, Strumpf NE, Nuamah IF, Adler DC, Cooley  ME, Jepson C, et al. A specialized home care intervention  improves survival among older post-surgical cancer patients. *Journal of the American Geriatrics Society* 2000;**48**(12):  1707–13. |  | x |  |  |  |  |  |  |  |  |  | 1 |
| McCorkle R, Dowd M, Ercolano E, Schulman-Green D, Williams AL, Siefert ML, et al. Effects of a nursing intervention on quality of life outcomes in post-surgical women with gynecological cancers. *Psycho-Oncology* 2009; **18**(1):62–70. |  | x |  |  |  |  |  |  |  |  |  | 1 |
| McDonald MV, Pezzin LE, Feldman PH, Murtaugh CM, Peng TR. Can just-in-time, evidence-based “reminders” improve pain management among home health care nurses and their patients? *Journal of Pain and Symptom* *Management* 2005;**29**(5):474–88. |  | x |  |  |  |  |  |  |  |  |  | 1 |
| McGillis Hall, L., Doran, D., Pink, G.H., 2004. Nurse staffing models, nursing hours, and patient safety outcomes. J. Nurs. Adm. 34 (1), 41–45. |  |  |  |  |  |  |  |  |  |  | x | 1 |
| McInnes E, Mira M, Atkin N, Kennedy P, Cullen J: Can GP input into discharge planning result in better outcomes for the frail aged: results from a randomized controlled trial. Fam Pract 1999, 16(3):289–293. | x |  |  |  |  |  |  |  |  |  |  | 1 |
| McKegney FP, Bailey LR, Yates JW. Prediction and management of pain in patients with advanced cancer.  *General Hospital Psychiatry* 1981;**3**(2):95–101. |  | x |  |  |  |  |  |  |  |  |  | 1 |
| McLachlan SA, Allenby A, Matthews J, Wirth A, Kissane D, Bishop M, et al. Randomized trial of coordinated psychosocial interventions based on patient self-assessments versus standard care to improve the psychosocial functioning of patients with cancer. *Journal of Clinical Oncology* 2001;  **19**(21):4117–25. |  | x |  |  |  |  |  |  |  |  |  | 1 |
| McMullen, M., Alexander, M.K., Bourgeois, A., & Goodman, L. (2001). Evaluating a nurse practitioner service. *Dimensions*  *of Critical Care Nursing, 20*(5), 30-34. |  |  |  |  |  | x |  |  |  |  |  | 1 |
| McWhinney IR, Bass MJ, Donner A. Evaluation of a palliative care service: problems and pitfalls. *BMJ* 1994;**309** (6965):1340–2. |  | x |  |  |  |  |  |  |  |  |  | 1 |
| Meisinger C, Stollenwerk B, Kirchberger I, et al. Effects of a nurse-based case management compared to usual care among patients with myocardial infarction: results from the randomized controlled KORINNA study. BMC Geriatr 2013;13:115. |  |  |  |  |  |  |  |  |  | x |  | 1 |
| Melis RJ, van Eijken MI, Teerenstra S, van Achterberg T, Parker SG, Borm GF, et al. A randomized study of a multidisciplinary program to intervene on geriatric syndromes in vulnerable older people who live at home (Dutch EASY care Study). J Gerontol A Biol Sci Med Sci. 2008;63(3):283-90. |  |  |  |  | x |  |  |  |  |  |  | 1 |
| Menees SB, Patel DA, Dalton V: Colorectal cancer screening practices among obstetrician/gynecologists and nurse practitioners. J Women’s Health 2009, 18(8):1233–1238. |  |  |  |  |  |  |  |  | x |  |  | 1 |
| Meulepas MA, Braspenning JC, de Grauw WJ, et al. Patient-oriented intervention in addition to centrally organised checkups improves diabetic patient outcome in primary care. *Qual Saf Health Care*. 2008;17(5):324-8. |  |  |  |  |  |  |  | x |  |  |  | 1 |
| Meyer, S.C., & Miers, L.J. (2005). Cardiovascular surgeon and acute care nurse practitioner: Collaboration on postoperative outcomes. *AACN* *Advanced Critical Care, 16*(2), 149-158. |  |  |  |  |  | x |  |  |  |  |  | 1 |
| Miller, S.K. (1997). Impact of a gerontological nurse practitioner on the nursing home elderly in the acute care setting. *AACN Advanced Critical Care, 8*(4), 609-615. |  |  |  |  |  | x |  |  |  |  |  | 1 |
| Mills ME, Murray LJ, Johnston BT, Cardwell C, Donnelly M. Does a patient-held quality-of-life diary benefit patients with inoperable lung cancer?. *Journal of Clinical Oncology*  2009;**27**(1):70–7. |  | x |  |  |  |  |  |  |  |  |  | 1 |
| Mitchell GK, Del Mar CB, O’Rourke PK, Clavarino AM. Do case conferences between general practitioners and specialist palliative care services improve quality of life? A randomised controlled trial (ISRCTN 52269003). *Palliative Medicine* 2008;**22**(8):904–12. |  | x |  | x |  |  |  |  |  |  |  | 2 |
| Moher M, Yudkin P, Wright L, et al. Cluster randomised controlled trial to compare three methods of promoting secondary prevention of coronary heart disease in primary care. BMJ 2001;322:1338. |  |  |  |  |  |  |  |  |  | x |  | 1 |
| Moody NB, Smith PL, Glenn LL: Client characteristics and practice patterns of nurse practitioners and physicians. Nurse Pract 1999, 24(3):94–96. 99–100, 102–103. |  |  |  |  |  |  |  |  | x |  |  | 1 |
| Moore S, Corner J, Haviland J, Wells M, Salmon E, Normand C, et al. Nurse led follow up and conventional medical follow up in management of patients with lung cancer: randomised trial. *BMJ* 2002;**325**(7373):1145. |  | x |  |  |  |  |  |  |  |  |  | 1 |
| Mor V, Wool M, Guadagnoli E, Allen S. The impact of short term case management on cancer patients’ concrete needs and quality of life. *Advances in Medical Sociology* 1995;**6**:269–94. |  | x |  |  |  |  |  |  |  |  |  | 1 |
| Mundinger, M.O., Kane, R.L., Lenz, E.R., Totten, A.M., Tsai, W.Y., Cleary, P.D., Shelanski, M.L. (2000). Primary care outcomes in patients treated by nurse practitioners or physicians: A randomized trial. *Journal of the* *American Medical Association,* *283*(1), 59-68. |  |  | x |  |  | x |  |  |  |  |  | 2 |
| Murphy PA: Primary care for women. Health assessment, health promotion, and disease prevention services. J Nurse Midwifery 1996, 41(2):83–91. |  |  |  |  |  |  |  |  | x |  |  | 1 |
| Naglie G, Tansey C, Kirkland JL, Ogilvie-Harris DJ, Detsky AS, Etchells E, et al. Interdisciplinary inpatient care for elderly people with hip fracture: a randomized controlled trial. Cmaj. 2002;167(1):25-32. |  |  |  |  | x |  |  |  |  |  |  | 1 |
| Naji S, Cameron I, Russell I,Harvey R, LengM,McLeod K,  Murchison L, Pearson D, Philip F, Williams M. Integrated  care for diabetes: clinical, psychosocial, and economic  evaluation. Diabetes Integrated Care Evaluation Team.  *BMJ* 1994;**308**(6938):1208–12. |  |  |  |  |  |  | x |  |  |  |  | 1 |
| Naylor MD, Brooten DA, Campbell RL, Maislin G, McCauley KM, Schwartz JS: Transitional care of older adults hospitalized with heart failure: a randomized, controlled trial. J Am Geriatr Soc 2004, 52(5):675–684. | x |  |  |  |  |  |  |  |  |  |  | 1 |
| Naylor MD, Brooten D, Campbell R, Jacobsen BS, Mezey MD, Pauly MV, Schwartz JS: Comprehensive discharge planning and home follow-up of hospitalized elders. JAMA 1999, 281(7):613–620. | x |  |  |  |  |  |  |  |  |  |  | 1 |
| Naylor M, Brooten D, Jones R, Lavizzo-Mourey R, Mezey M, Pauly M: Comprehensive discharge planning for the hospitalized elderly. A randomized clinical trial. Ann Intern Med 1994, 120(12):999–1006. | x |  |  |  |  |  |  |  |  |  |  | 1 |
| Naylor MD: Comprehensive discharge planning for hospitalized elderly: a pilot study. Nurs Res 1990, 39(3):156–161. | x |  |  |  |  |  |  |  |  |  |  | 1 |
| Nelson, E.W., Van Cleve, S., Swartz, M.K., Kessen, W., & McCarthy, P.L. (1991). Improving the use of early follow-up  care after emergency department visits: A randomized trial. *Archives of* *Pediatrics and Adolescent Medicine,* *145*(4), 440-444. |  |  |  |  |  | x |  |  |  |  |  | 1 |
| New JP, Mason JM, Freemantle N, et al. Educational outreach in diabetes to encourage practice nurses to use primary care hypertension and hyperlipidaemia guidelines (EDEN): a randomized controlled trial. *Diabet Med*. 2004;21(6):599-603. |  |  |  |  |  |  |  | x |  |  |  | 1 |
| New JP, Mason JM, Freemantle N, et al. Specialist nurse-led intervention to treat and control hypertension and hyperlipidemia in diabetes (SPLINT): a randomized controlled  trial. *Diabetes Care*. 2003;26(8):2250-5. |  |  |  |  |  |  |  | x |  |  |  | 1 |
| Nikolaus T, Specht-Leible N, Bach M, Oster P, Schlierf G. A randomized trial of comprehensive geriatric assessment and home intervention in the care of hospitalized patients. Age Ageing. 1999;28(6): 543-50. |  |  |  |  | x |  |  |  |  |  |  | 1 |
| Nilasena DS, Lincoln MJ. A computer-generated reminder  system improves physician compliance with diabetes  preventive care guidelines. Proceedings-the Annual  Symposium on Computer Applications in Medical Care.  1995:640–5. |  |  |  |  |  |  | x |  |  |  |  | 1 |
| Oakley, D., Murray, M.E., Murtland, T., Hayashi, R., Frank Andersen, H., Mayes, F., & Rooks, J. (1996). Comparisons of outcomes of maternity care by obstetricians and certified nurse midwives. *Obstetrics & Gynecology,* *88*(5), 823-829. |  |  |  |  |  | x |  |  |  |  |  | 1 |
| Oakley, D., Murtland, T., Mayes, F., Hayashi, R., Petersen, B. A., Rorie, C., & Andersen, F. (1995). Processes of care: Comparisons of certified nurse midwives and obstetricians. *Journalof Nurse-Midwifery, 40*(5), 399-409. |  |  |  |  |  | x |  |  |  |  |  | 1 |
| O’Connor PJ, Rush WA, Peterson J, Morben P, Cherney L,  Keogh C, Lasch S. Continuous quality improvement can  improve glycemic control for HMO patients with diabetes.  *Archives of Family Medicine* 1996;**5**(9):502–6. |  |  |  |  |  |  | x |  |  |  |  | 1 |
| O’Hare JP, Raymond NT, Mughal S, et al. Evaluation of delivery of enhanced diabetes care to patients of South Asian ethnicity: the United Kingdom Asian Diabetes Study (UKADS). *Diabet Med*. 2004;21(12):1357-65. |  |  |  |  |  |  |  | x |  |  |  | 1 |
| Oleske DM, Hauck WW. A population-based evaluation of the impact of interventions for improving care to cancer patients in the home setting. *Home Health Care Services* *Quarterly* 1988;**9**(1):45–61. |  | x |  |  |  |  |  |  |  |  |  | 1 |
| Oliveria SA, Altman JF, Christos PJ, Halpern AC: Use of nonphysician health care providers for skin cancer screening in the primary care setting. Prev Med 2002, 34(3):374–379. |  |  |  |  |  |  |  |  | x |  |  | 1 |
| Paez, K.A., & Allen, J.K. (2006). Cost-effectiveness of nurse practitioner management of hypercholesterolemia following  coronary revascularization. *Journal of the American Academy of* *Nurse Practitioners, 18*(9), 436-444. |  |  |  |  |  | x |  |  |  |  |  | 1 |
| Palmer RH, Louis TA, Hsu LN, Peterson HF, Rothrock  JK, Strain R, Thompson MS, Wright EA. A randomized  controlled trial of quality assurance in sixteen ambulatory  care practices. *Medical Care* 1985;**23**(6):751–70. |  |  |  |  |  |  | x |  |  |  |  | 1 |
| Patrician, P.A., Loan, L., McCarthy, M., Fridman, M., Donaldson, N., Bingham, M., Brosch, L.R., 2011. The association of shift-level nurse staffing with adverse patient events. J. Nurs. Adm. 41 (2), 64–70. |  |  |  |  |  |  |  |  |  |  | x | 1 |
| Patton LL, Ashe TE, Elter JR, Southerland JH, Strauss RP: Adequacy of training in oral cancer prevention and screening as self-assessed by physicians, nurse practitioners, and dental health professionals. Oral Surg Oral Med Oral Pathol Oral Radiol Endod 2006, 102(6):758–764. |  |  |  |  |  |  |  |  | x |  |  | 1 |
| Paul, S. (2000). Impact of a nurse-managed heart failure clinic: A pilot study. *American Journal of Critical Care,* *9*(2), 140-146. |  |  |  |  |  | x |  |  |  |  |  | 1 |
| Peters AL, Davidson MB. Application of a diabetes  managed care program. The feasibility of using nurses and  a computer system to provide effective care. *Diabetes Care*  1998;**21**(7):1037–43. |  |  |  |  |  |  | x |  |  |  |  | 1 |
| Philis-Tsimikas A, Walker C, Rivard L, et al. Improvement in diabetes care of underinsured patients enrolled in project dulce: a community-based, culturally appropriate, nurse case management and peer education diabetes care model. *Diabetes* *Care*. 2004;27(1):110-5. |  |  |  |  |  |  |  | x |  |  |  | 1 |
| Pieber TR, Holler A, Siebenhofer A, Brunner GA, Semlitsch  B, Schattenberg S. Evaluation of a structured teaching and  treatment programme for type 2 diabetes in general practice  in a rural area of Austria. *Diabetes Medicine* 1995;**12**(4):  349–54. |  |  |  |  |  |  | x |  |  |  |  | 1 |
| Pill R, Stott NCH, Rollnick SR, Rees M. A randomized  controlled trial of an intervention designed to improve  the care given in general practice to Type II diabetic  patients: patient outcomes and professional ability to  change behaviour. *Family Practice* 1998;**15**(3):229–35. |  |  |  |  |  |  | x |  |  |  |  | 1 |
| Pinkerton, J.A., & Bush, H.A. (2000). Nurse practitioners and physicians: Patients’ perceived health and satisfaction with  care. *Journal of the American Academy of Nurse Practitioners,*  *12*(6), 211-217. |  |  |  |  |  | x |  |  |  |  |  | 1 |
| Pioro, M.H., Landefeld, C.S., Brennan, P.F., Daly, B., Fortinsky, R.H., Kim, U., & Rosenthal, G.E. (2001). Outcomesbased  trial of an inpatient nurse practitioner service for general medical patients. *Journal of Evaluation in* *Clinical Practice, 7*(1), 21-33. |  |  |  |  |  | x |  |  |  |  |  | 1 |
| Preen DB, Bailey BES, Wright A, Kendall P, Phillips M, Hung J, Hendriks R, Mather A, Williams E: Effects of a multidisciplinary, post-discharge continuance of care intervention on quality of life, discharge satisfaction, and hospital length of stay: a randomized controlled trial. Int J Qual Health Care 2005, 17(1):43–51. | x |  |  |  |  |  |  |  |  |  |  | 1 |
| Price JH, Jordan TR, Dake JA: Perceptions and use of smoking cessation in nurse-midwives’ practice. J Midwifery Womens Health 2006, 51(3):208–215. |  |  |  |  |  |  |  |  | x |  |  | 1 |
| Purdy, N., Spence Laschinger, H.K., Finegan, J., Kerr, M., Olivera, F., 2010. Effects of work environments on nurse and patient outcomes. J. Nurs. Manag. 18 (8), 901–913. |  |  |  |  |  |  |  |  |  |  | x | 1 |
| Rao AV, Hsieh F, Feussner JR, Cohen HJ. Geriatric evaluation and management units in the care of the frail elderly cancer patient 3874. *Journals of Gerontology Series A -* *Biological Sciences & Medical Sciences* 2005;**60**(6):798–803. |  | x |  |  |  |  |  |  |  |  |  | 1 |
| Rawl SM, Given BA, Given CW, Champion VL, Kozachik SL, Barton D, et al. Intervention to improve psychological functioning for newly diagnosed patients with cancer. *Oncology Nursing Forum* 2002;**29**(6):967–75. |  | x |  |  |  |  |  |  |  |  |  | 1 |
| Rideout, K. (2007). Evaluation of a PNP care coordinator model for hospitalized children, adolescents, and young adults with cystic fibrosis. *Pediatric* *Nursing, 33*(1), 29-35. |  |  |  |  |  | x |  |  |  |  |  | 1 |
| Rith-Najarian S, Branchaud C, Beaulieu O, Gohdes  D, Simonson G, Mazze R. Reducing lower-extremity  amputations due to diabetes. Application of the staged  diabetes management approach in a primary care setting.  *Journal of Family Practice* 1998;**47**(2):127–32. |  |  |  |  |  |  | x |  |  |  |  | 1 |
| Ritz LJ, Nissen MJ, Swenson KK, Farrell JB, Sperduto PW,  Sladek ML, et al. Effects of advanced nursing care on quality of life and cost outcomes of women diagnosed with breast cancer. *Oncology Nursing Forum* 2000;**27**(6):923–32. |  | x |  |  |  |  |  |  |  |  |  | 1 |
| Robinson, J.N., Norwitz, E.R., Cohen, A.P., & Lieberman, E. (2000). Predictors of episiotomy use at first spontaneous vaginal delivery. *Obstetrics &* *Gynecology, 96*(2), 214-218. |  |  |  |  |  | x |  |  |  |  |  | 1 |
| Rosenblatt, R.A., Dobie, S.A., Hart, L.G., Schneeweiss, R., Gould, D., Raine, T. R. Perrin, E.B. (1997). Inter-specialty differences in the obstetric care of low-risk women. *American*  *Journal of Public Health, 87*(3), 344-351. |  |  |  |  |  | x |  |  |  |  |  | 1 |
| Rudd P, Miller NH, Kaufman J, et al. Nurse management for hypertension. A systems approach. *Am J Hypertens*. 2004;17(10):921-7. |  |  |  |  |  |  |  | x |  |  |  | 1 |
| Ruiz, R. J., Brown, C. E., Peters, M.T., & Johnson, A.B. (2001). Specialized care for twin gestations: Improving new - born outcomes and reducing costs. *Journal of Obstetric, Gynecologic and* *Neonatal Nursing, 30*, 52-60. |  |  |  |  |  | x |  |  |  |  |  | 1 |
| Running A, Kipp C, Mercer V: Prescriptive patterns of nurse practitioners and physicians. J Am Acad Nurse Pract 2006, 18(5):228–233. |  |  |  |  |  |  |  |  | x |  |  | 1 |
| Russell, D., VorderBruegge, M., & Burns, S.M. (2002). Effect of an outcomesmanaged approach to care of neuroscience  patients by acute care nurse practitioners. *American Journal of* *Critical Care, 11*(4), 353-362. |  |  |  |  |  | x |  |  |  |  |  | 1 |
| Rutherford A, Burge B. General practitioners and hospitals.  Continuity of care. *Australian Family Physician* 2001;**30**  (11):1101–7. |  | x |  |  |  |  |  |  |  |  |  | 1 |
| Rutten G, van Eijk J, de Nobel E, Beek M, van der Velden  H. Feasibility and effects of a diabetes type II protocol with  blood glucose self-monitoring in general practice. *Family*  *Practice* 1990;**7**(4):273–8. |  |  |  |  |  |  | x |  |  |  |  | 1 |
| Sadur CN, Moline N, Costa M, Michalik D, Mendlowitz  D, Roller S. Diabetes management in a health maintenance  organization. Efficacy of care management using cluster  visits. *Diabetes Care* 1999;**22**(12):2011–7. |  |  |  |  |  |  | x |  |  |  |  | 1 |
| Sansbury LB, Klabunde CN, Mysliwiec P, Brown ML: Physicians’ use of nonphysician healthcare providers for colorectal cancer screening. Am J Prev Med 2003, 25(3):179–186. |  |  |  |  |  |  |  |  | x |  |  | 1 |
| Schraeder C, Shelton P, Sager M. The effects of a collaborative model of primary care on the mortality and hospital use of community-dwelling older adults. J Gerontol A Biol Sci Med Sci. 2001;56(2): M106-12. |  |  |  |  | x |  |  |  |  |  |  | 1 |
| Schultz, J.M., Liptak, G.S., & Fioravanti, J. (1994). Nurse practitioners’ effectiveness in NICU. *Nursing Management,*  *25*(10), 50-53. |  |  |  |  |  | x |  |  |  |  |  | 1 |
| Seago, J.A., Williamson, A., Atwood, C., 2006. Longitudinal analyses of nurse staffing and patient outcomes: more about failure to rescue. J. Nurs. Adm. 36 (1), 13–21. |  |  |  |  |  |  |  |  |  |  | x | 1 |
| Shaheen NJ, Crosby MA, O’Malley MS, Murray SC, Sandler RS, Galanko JA, Ransohoff DF, Klenzak JS: The practices and attitudes of primary care nurse practitioners and physician assistants with respect to colorectal cancer screening. Am J Gastroenterol 2000, 95(11):3259–3265. |  |  |  |  |  |  |  |  | x |  |  | 1 |
| Shuldham, C., Parkin, C., Firouzi, A., Roughton, M., Lau-Walker, M., 2009. The relationship between nurse staffing and patient outcomes: a case study. Int. J. Nurs. Stud. 46 (7), 986–992. |  |  |  |  |  |  |  |  |  |  | x | 1 |
| Shultz EK, Bauman A, Hayward M, Holzman R. Improved  care of patients with diabetes through telecommunications.  *Ann of the New York Academy of Sciences* 1992;**670**:141–5. |  |  |  |  |  |  | x |  |  |  |  | 1 |
| Skrutkowski M, Saucier A, Eades M, Swidzinski M, Ritchie J, Marchionni C, et al. Impact of a pivot nurse in oncology on patients with lung or breast cancer: symptom distress, fatigue, quality of life, and use of healthcare resources. *Oncology Nursing Forum* 2008;**35**(6):948–54. |  | x |  |  |  |  |  |  |  |  |  | 1 |
| Smith DM, Norton JA,Weinberger M,McDonald CJ, Katz  BP. Increasing prescribed office visits. A controlled trial in  patients with diabetes mellitus. *Medical Care* 1986;**24**(3):  189–99. [MEDLINE: 86145682]  Smith DM, Weinberger M, Katz BP. A controlled trial to  increase office visits and reduce hospitalizations of diabetic  patients. *Journal of General Internal Medicine* 1987;**2**(4):  232–8. |  |  |  |  |  |  | x |  |  |  |  | 1 |
| Sommers LS, Marton KI, Barbaccia JC, Randolph J. Physician, nurse, and social worker collaboration in primary care for chronically seniors. Arch Intern Med. 2000;160(12):1825-33. |  |  |  |  | x |  |  |  |  |  |  | 1 |
| Stein GH. The use of a nurse practitioner in the  management of patients with diabetes mellitus. *Medical*  *Care* 1974;**12**(10):885–90. |  |  |  |  |  |  | x |  |  |  |  | 1 |
| Stone, P.W., Mooney-Kane, C., Larson, E.L., Horan, T., Glance, L.G., Zwanziger, J., Dick, A.W., 2007. Nurse working conditions and patient safety outcomes. Med. Care 45 (6), 571–578. |  |  |  |  |  |  |  |  |  |  | x | 1 |
| Stuck, A.E., Aronow, H.U., Steiner, A., Alessi, C.A., Bula, C.J., Gold, M.N., Beck, J.C. (1995). A trial of annual inhome  comprehensive geriatric assessments for elderly people living in the community. *New England Journal of* *Medicine, 333*(18), 1184-1189. |  |  |  |  |  | x |  |  |  |  |  | 1 |
| Sullivan FM, Menzies A. The costs and benefits of  introducing a nurse-run diabetic review service into general  practice. *Practical Diabetes* 1991;**8**(2):47–50. |  |  |  |  |  |  | x |  |  |  |  | 1 |
| Sze, E.H., Ciarleglio, M., & Hobbs, G. (2008). Risk factors associated with anal sphincter tear difference among midwife, private obstetrician, and resident deliveries. *International Uro -gynecology Journal, 19*(8), 1141-1144. |  |  |  |  |  | x |  |  |  |  |  | 1 |
| Tai SS, Nazareth I, Donegan C, Haines A. Evaluation of  general practice computer templates: Lessons from a pilot  randomised controlled trial. *Methods of Information in*  *Medicine* 1999;**38**(3):177–81. |  |  |  |  |  |  | x |  |  |  |  | 1 |
| Taplin S, Galvin MS, Payne T, Coole D, Wagner E. Putting  population-based care into practice: real option or rhetoric.  *Journal of the American Board of Family Practice* 1998;**11**(2):  116–26. |  |  |  |  |  |  | x |  |  |  |  | 1 |
| Taylor CB, Miller NH, Reilly KR, et al. Evaluation of a nurse-care management system to improve outcomes in patients with complicated diabetes. *Diabetes Care*. 2003;26(4):1058-63. |  |  |  |  |  |  |  | x |  |  |  | 1 |
| Taylor, J.A., Dominici, F., Agnew, J., Gerwin, D., Morlock, L., Miller, M.R., 2012. Do nurse and patient injuries share common antecedents? An analysis of associations with safety climate and working conditions. BMJ Qual. Saf. 21, 101–111. |  |  |  |  |  |  |  |  |  |  | x | 1 |
| Temel JS, Greer JA, Muzikansky A, Gallagher ER, Admane S, Jackson VA, et al. Early palliative care for patients with metastatic non-small-cell lung cancer. N Engl J Med. 2010;363(8):733-42. |  |  |  | x |  |  |  |  |  |  |  | 1 |
| Titler, M.G., Shever, L.L., Kanak, M.F., Picone, D.M., Qin, R., 2011. Factors associated with falls during hospitalization in an older adult population. Res. Theory Nurs. Pract. 25 (2), 127–148. |  |  |  |  |  |  |  |  |  |  | x | 1 |
| Tompkins TH, Belza B, Brown MA: Nurse practitioner practice patterns for exercise counseling. J Am Acad Nurse Pract 2009, 21(2):79–86. |  |  |  |  |  |  |  |  | x |  |  | 1 |
| Trowbridge R, Dugan W, Jay SJ, Littrell D, Casebeer LL, Edgerton S, et al. Determining the effectiveness of a clinical-practice intervention in improving the control of pain in outpatients with cancer. *Academic Medicine* 1997;  **72**(9):798–800. |  | x |  |  |  |  |  |  |  |  |  | 1 |
| Twigg, D., Duffield, C., Bremner, A., Rapley, P., Finn, J., 2011. The impact of the nursing hours per patient day (NHPPD) staffing method on patient outcomes: a retrospective analysis of patient and staffing data. Int. J. Nurs. Stud. 48 (5), 540–548. |  |  |  |  |  |  |  |  |  |  | x | 1 |
| Unruh, L.Y., Zhang, N.J., 2012. Nurse staffing and patient safety in hospitals: new variable and longitudinal approaches. Nurs. Res. 61 (1), 3–12. |  |  |  |  |  |  |  |  |  |  | x | 1 |
| Vallieres I, Aubin M, Blondeau L, Simard S, Giguere A.  Effectiveness of a clinical intervention in improving pain control in outpatients with cancer treated by radiation therapy. *International Journal of Radiation Oncology, Biology,*  *Physics* 2006;**66**(1):234–7. |  | x |  |  |  |  |  |  |  |  |  | 1 |
| Van den Heede, K., Sermeus, W., Diya, L., Clarke, S.P., Lesaffre, E., Vleugels, A., Aiken, L.H., 2009. Nurse staffing and patient outcomes in Belgian acute hospitals: cross-sectional analysis of administrative data. Int. J. Nurs. Stud. 46 (7), 928–939. |  |  |  |  |  |  |  |  |  |  | x | 1 |
| Varughese, A.M., Byczkowski, T.L., Wittkugel, E.P., Kotagal, U., & Dean, K. (2006). Impact of a nurse practitioner assisted  preoperative assessment program on quality. *Pediatric Anesthesia,* *16*(7), 723-733. |  |  |  |  |  | x |  |  |  |  |  | 1 |
| Velikova G, Booth L, Smith AB, Brown PM, Lynch P, Brown JM, et al. Measuring quality of life in routine oncology practice improves communication and patient well-being: A randomized controlled trial. *Journal of* *Clinical Oncology* 2004;**22**(4):714–24. |  | x |  |  |  |  |  |  |  |  |  | 1 |
| Vinicor F, Cohen SJ, Mazzuca SA, Moorman N, Wheeler  M, Kuebler T. DIABEDS: a randomized trial of the effects  of physician and/or patient education on diabetes patient  outcomes. *Journal of Chronic Diseases* 1987;**40**(4):345–56. |  |  |  |  |  |  | x |  |  |  |  | 1 |
| Voogdt-Pruis HR, Beusmans GH, Gorgels AP, et al. Effectiveness of nurse-delivered cardiovascular risk management in primary care: a randomised trial. Br J Gen Pract 2010;60:40–6. |  |  |  |  |  |  |  |  |  | x |  | 1 |
| Walders N, Kercsmar C, Schluchter M, Redline S, Kirchner HL, Drotar D. An interdisciplinary intervention for undertreated pediatric asthma. Chest. 2006;129(2):292-9. |  |  |  |  | x |  |  |  |  |  |  | 1 |
| Wallymahmed ME, Morgan C, Gill GV, et al. Nurse-led cardiovascular risk factor intervention leads to improvements in cardiovascular risk targets and glycaemic control in people with Type 1 diabetes when compared with routine diabetes clinic attendance. *Diabet Med*. 2011;28(3):373-9. |  |  |  |  |  |  |  | x |  |  |  | 1 |
| Ward A, Kamien M, Mansfield F, Fatovich B. Educational  feedback in the management of type 2 diabetes in general  practice. *Educ Gen Pract* 1996;**7**:142–50. |  |  |  |  |  |  | x |  |  |  |  | 1 |
| Wattchow DA, Weller DP, Esterman A, Pilotto LS, McGorm K, Hammett Z, et al. General practice vs surgicalbased follow-up for patients with colon cancer: randomized controlled trial. *British Journal of Cancer* 2006;**94**(8):1116–21. |  | x |  |  |  |  |  |  |  |  |  | 1 |
| Weinberger M, Kirkman MS, Samsa GP, Shortliffe EA,  Landsman PB, Cowper PA, Simel DL, Feussner JR. A  nurse-coordinated intervention for primary care patients  with non-insulin-dependent diabetes mellitus: impact on  glycemic control and health-related quality of life. *Journal of*  *General Internal Medicine* 1995;**10**(2):59–66. |  |  |  |  |  |  | x |  |  |  |  | 1 |
| Weinberger M, Oddone EZ, Henderson WG: Does increased access to primary care reduce hospital readmissions? New Engl J Med 1996, 334(22):1441–1447. | x |  |  |  |  |  |  |  |  |  |  | 1 |
| Wells NHJT. Improving cancer pain management through  patient and family education. *Journal of Pain and Symptom*  *Management* 2003;**25**(4):344–56. |  | x |  |  |  |  |  |  |  |  |  | 1 |
| Wells M, Harrow A, Donnan P, Davey P, Devereux S, Little  G, et al. Patient, carer and health service outcomes of nurseled  early discharge after breast cancer surgery: a randomised  controlled trial. *British Journal of Cancer* 2004;**91**(4):651–8. |  | x |  |  |  |  |  |  |  |  |  | 1 |
| West CM, Dodd MJ, Paul SM, Schumacher K, Tripathy D, Koo P, et al. The PRO-SELF(c): Pain Control Program—an effective approach for cancer pain management. *Oncology* *Nursing Forum* 2003;**30**(1):65–73. |  | x |  |  |  |  |  |  |  |  |  | 1 |
| Williams JG, Cheung WY, Chetwynd N, Cohen DR, El-  Sharkawi S, Finlay I, et al. Pragmatic randomised trial to  evaluate the use of patient held records for the continuing  care of patients with cancer. *Quality in Health Care* 2001;  **10**(3):159–65. |  | x |  |  |  |  |  |  |  |  |  | 1 |
| Wolf, D., Lehman, L., Quinlin, R., Rosenzweig, M., Friede, S., Zullo, T., Hoffman, L., 2008. Can nurses impact patient outcomes using a patient-centered care model? J. Nurs. Adm. 38 (12), 532–540. |  |  |  |  |  |  |  |  |  |  | x | 1 |
| Wood DA, Kotseva K, Connolly S, et al. Nurse-coordinated multidisciplinary, family-based cardiovascular disease prevention programme (EUROACTION) for patients with coronary heart disease and asymptomatic individuals at high risk of cardiovascular disease: a paired, cluster-randomised controlled trial. Lancet 2008;371:1999–2012. |  |  |  |  |  |  |  |  |  | x |  | 1 |
| Young W, Rewa G, Goodman SG, et al. Evaluation of a community-based inner-city disease management program for postmyocardial infarction patients: a randomized  controlled trial. CMAJ 2003;169:905–10. |  |  |  |  |  |  |  |  |  | x |  | 1 |
| Zahnd EG, Coates TJ, Richard RJ, Cummings SR: Counseling medical patients about cigarette smoking: a comparison of the impact of training on nurse practitioners and physicians. Nurse Pract 1990, 15(3):10–13. 17–18. |  |  |  |  |  |  |  |  | x |  |  | 1 |
| Zimmermann C, Swami N, Krzyzanowska M, Hannon B, Leighl NB, Oza AM, et al. Early palliative care for patients with advanced cancer: a cluster-randomised controlled trial. The Lancet. 2014;383 (9930):1721-30. |  |  |  | x |  |  |  |  |  |  |  | 1 |
